# Bat Cutaneous Microbial Assemblage Functional Redundancy Across a Host-Mediated Disturbance

**DOI:** 10.1007/s00248-024-02480-2

**Published:** 2024-12-21

**Authors:** Matthew Grisnik, Donald M. Walker

**Affiliations:** 1 Department of Biology, Middle Tennessee State University, Murfreesboro, TN 37132 USA; 2 Department of Biology, Coastal Carolina University, Conway, SC 29528 USA

**Keywords:** Microbiome, *Eptesicus fuscus*, Community assembly, Host-microbe interactions, Microbial resilience

## Abstract

Understanding the processes and factors that influence the structure of host-associated microbial assemblages has been a major area of research as these assemblages play a role in host defense against pathogens. Previous work has found that bacterial taxa within bat cutaneous microbial assemblages have antifungal capabilities against the emerging fungal pathogen, *Pseudogymnoascus destructans.* However, our understanding of natural fluctuations in these cutaneous microbial assemblages over time due to shifts in host habitat is lacking. The objective of this work was to understand how the taxonomic and functional bat cutaneous microbial assemblage responds to seasonal shifts in host habitat. We hypothesized that at the community level, there will be turnover in taxonomic structure but functional redundancy across seasons. On a finer scale, we hypothesized that there will be differences in the relative abundance of functional genes that code for select pathways across seasons. Results showed that, on a broad scale, the bat cutaneous microbial assemblage is seasonally taxonomically dynamic but functionally redundant. Additionally, although there was almost complete taxonomic turnover between winter and summer bat microbial assemblages, there was no difference in assemblage structure across winters. This functional redundancy was also observed at finer scales, with no differences in the abundance of genes within pathways of hypothesized importance across seasons or winters. Taken together, results suggest species sorting mechanisms correlated with shifts in host habitat use, drive taxonomic but not functional host-associated cutaneous microbial community assembly.

**Supplementary Information:**

The online version contains supplementary material available at 10.1007/s00248-024-02480-2.

## Introduction

Elucidating the drivers of host-associated microbial community assembly is vital given these assemblages’ hypothesized role in maintaining host health [1, 2]. Previous work suggests that rapid changes in the structure of host-associated cutaneous microbial assemblages are associated with dysbiosis and declines in host health [3–5]. Therefore, understanding the stability of these assemblages over spatiotemporal scales is important for our understanding of the microbiome’s role in host defense. Previous work has shown that host-associated cutaneous microbial assemblage structure is correlated with a variety of factors including host identity [6], host life history [7], environmental factors [8–10], host social behavior [11], and disease state [12, 13]. Additionally, time has been shown to be a significant factor, with previous work on bat cutaneous microbial assemblages showing that samples collected on the same day across sites are more similar than samples from the same individual across days [14]. However, overall, little work has been done to understand how host seasonal behavior influences cutaneous microbial assemblages along a temporal scale.

Understanding the processes that maintain these microbial assemblage’s structure over time is a central goal of microbial ecology, and understanding how these processes change in the presence of a disturbance is key to understanding its role in host–pathogen defense. Overall, microbial assemblages can be said to respond to disturbance with resistance, resilience, and/or functional redundancy [15]. An assemblage can be described as resistant if the structure does not change, whereas resilience is the ability to return to a pre-disturbance state [16]. Lastly, functional redundancy is defined as the ability of a community to maintain its original functions despite a change in its taxonomic composition [15]. The process driving patterns of functional redundancy in microbial assemblages is still uncertain. Previous work has suggested two potential drivers, the first is a lottery hypothesis which states that an assemblage is colonized randomly from a pool of functionally equivalent species [17, 18]. In this hypothesis, environmental selection or species sorting, occurs on the functional aspect of microbial taxa. Alternatively, the species sorting mechanism works on taxonomy alone, and the bacterial taxa are functionally equivalent [15, 19]. Currently, our understanding of functional redundancy within host-associated microbial assemblages is primarily based on comparisons of functional pathways that represent essential cell functions, such as metabolism and amino acid synthesis [20]. However, few studies have incorporated finer-scale observations, specifically observing variation in functional pathways of hypothesized importance across taxonomic differences.

Previous work on host-associated microbial assemblage function has mainly focused on the interaction between a bacterial microbiome and a fungal pathogen. Functional pathways that are hypothesized to be of importance in interactions between pathogens and host-associated cutaneous microbial assemblages include membrane transport, biosynthesis of secondary metabolites, and the metabolism of terpenoid and polyketides [21]. It is suggested that these pathways play a role in both bacterial communication, as well as responding to external stimuli such as pathogen invasion [21, 22] across host species. Interestingly, recent work has found that the presence of *Pseudogymnoascus destructans* (*Pd*), the fungal agent of white-nose syndrome in bats, was correlated with a higher abundance of genes within these pathways [23], suggesting an interaction between pathogen and the structuring processes of these microbial assemblages. While less studied in host-associated microbial assemblages, microbial functions associated with other processes such as metabolism, can reveal interesting shifts in available energy sources. For example, in studies of microbial assemblages in marine soil sediment, it was found that soils taken from the surface were more enriched with photosynthetic bacterial taxa [24]. While previous work has primarily focused on understanding interactions between cutaneous microbial assemblages and fungal pathogens, little work has been done to understand natural fluctuations in cutaneous microbial assemblage structure due to host-mediated behaviors such as seasonal shifts in host habitat usage.

Since its introduction into the USA in 2006, *Pd* has rapidly spread killing millions of bats and threatening multiple bat species with extinction [25, 26]. The emergence of this pathogen has led to a number of studies that use this system to understand host-microbiome-pathogen interactions [12, 13, 27, 28]; however, understanding the influence of natural host behavior on the bat cutaneous microbial assemblage is lacking. The Big Brown Bat, *Eptesicus fuscus,* is one of the widest-ranging mammals in North America and is often described as a habitat generalist [29]. During the summer, *E. fuscus* uses a variety of habitats and will roost in both natural (i.e., caves, tree cavities) and anthropogenic structures (i.e., buildings; [29]). However, during the winter hibernation season, the majority of *E. fuscus* remain in caves for long periods of time. The effects of this seasonal shift in habitat use on bat cutaneous microbial assemblages have not been addressed, with most studies only looking at bat cutaneous microbial assemblages during the winter hibernation season.

The objective of this study was to understand how the host cutaneous microbial assemblage responds to seasonal shifts in host habitat both taxonomically and functionally. Specifically, we aimed to (1) determine if the taxonomic composition of the host cutaneous microbial assemblage changes during the seasonal shift in host habitat, as well as, across years, (2) determine if there is a difference in putative bacterial microbiome function across seasons and years, and (3) determine if there are fine-scale shifts in gene abundance across seasons. We hypothesized that there will be broad-scale functional redundancy but taxonomic variability across seasons but not years. Additionally, at finer scales, we hypothesized that there will be seasonal differences in the relative abundance of functional genes that code for select pathways.

## Methods

### Sample Collection

Swabs from 57 individual adult *Eptesicus fuscus* were collected during statewide surveys between January 2017 and July 2018 across 26 sites in Tennessee. Sites were defined as either individual caves in winter or as trapping locations in the summer, and trapping was done at multiple wildlife management areas (Supplemental Table C1). To remove the influence of the site on our data, when sites had more than one sample, we randomly selected one bat from each site for downstream analysis. This resulted in a total of 26 individual bats from 26 unique sites (Supplemental Table C1). Specifically, 6 samples were collected during the winter of 2017, 2 samples from the summer of 2017, 14 samples from the winter of 2018, and 4 samples from the summer of 2018. Small sample sizes presented here are due to a combination of low catch rate of the species within sites, and selection of samples that were *Pd* negative. Each bat was sampled following the protocol outlined in Grisnik [13]. More specifically, each bat had five swab strokes (sterile Puritan polyester tipped swabs, Puritan, Guilford Maine) taken from their muzzles/external ears (avoiding their mouths), and an additional five swab strokes taken from wings and fur, using one swab per bat. All samples were stored on ice in the field until they could be stored permanently at − 80 °C.

We extracted DNA from the 26 swabs using a Qiagen DNeasy PowerSoil HTP 96 kit following the manufacturer’s protocol. Each 96-well extraction plate included a single DNA extraction blank to allow us to filter out kit-based contamination during bioinformatics. Additionally, the 96-well extraction plate was set up, with the locations randomized to reduce potential bias in the well-to-well contamination [30]. After the final step of extraction, DNA was concentrated to ~ 25 µL using an Eppendorf Vacufuge plus.

### Microbial Assemblage Characterization

After extraction, we used next-generation sequencing to characterize the microbial assemblage of each bat sample. To reduce environmental as well as potential cross-contamination, each step in the library preparation protocol (DNA isolation, PCR setup, and post-PCR processes) was performed in a separate PCR cabinet. Each hood had its own designated set of pipettes that were autoclaved and UV crosslinked periodically throughout the process. To prepare the library, we followed a modified version of the Illumina 16S Metagenomics Sequencing Library Preparation protocol. We targeted the V4 region of the 16S rRNA marker using the primers 806R/515F [31]. Each PCR reaction contained 12.5 µL MCLAB I-5 Hi-Fi taq mastermix, 1 µL of 806R (10 µM), 1 µL of 515F (10 µM), 5.5 µL PCR grade water, and 5 µL DNA template. PCR amplification was performed with an initial denaturation at 95 °C for 2 min, followed by 35 cycles of 98 °C for 10 s, 55 °C for 15 s, and 72 °C for 5 s, with a final extension cycle of 72 °C for 5 min. After amplicon PCR and indexing steps, we used MAGBIO High-prep magnetic beads to clean PCR products. We quantified samples with a Promega Quantus Fluorometer, normalized, and pooled at a 4 picomolar concentration. The pooled library was then loaded onto an Illumina MiSeq v2 flow cell and sequenced using a 500-cycle reagent kit (PE 2 × 250 bp reads).

### Bioinformatics Processing

We processed amplicon sequencing reads using mothur v1.42.1 [32]. A total of 48,442,995 raw data sequence reads were obtained. Paired-end reads were assembled into contigs, sequences containing homopolymers (> 8 nucleotides) or any ambiguous base calls were removed. We then identified unique sequences and aligned them to the SILVA v123 bacterial reference database [33]. After alignment, sequences were trimmed to the V4 region and *pre-clustered* for two nucleotide differences between clusters. We used the *vsearch* [34] command in mothur to remove chimeras. We then removed sequences that were classified as Archaea, Eukaryota, chloroplast, mitochondria, or as unknown. We used the *cluster.split* command in mothur to cluster sequences into operational taxonomic units (OTUs) at 97% similarity [35]. Rare OTUs, those that appeared less than five times, were removed from the dataset. Additionally, OTUs that were found within the DNA extraction blanks were also removed (*n* = 1,669 OTUs). We selected OTUs as the focal taxonomic unit rather than ASVs (amplicon sequence variants) due to previous work that has shown that there is little difference in ecological patterns observed when ASV vs OTUs are used [36]. After all quality control steps were completed, we were left with 5,701,307 sequences (11.7% of the total). In order to account for a significant difference in library sizes across samples (Kruskal–Wallis: *χ*^2^ = 83.98, *p* < 0.05), we rarefied by subsampling each library at 1,200 sequence reads. This decision to subsample was based on previous work that has shown that subsampling is an effective way to account for variation in library size [37]. Our final OTU x sample matrix comprised 26 samples from *E. fuscus*. All mothur commands are included in the Supplemental File A for reproducibility purposes.

### Statistical Analyses

Prior to analyzing data, all OTUs that summed to less than two were removed to prevent undue weighing of these taxa in the presence/absence matrices discussed below, resulting in a total of 2,575 OTUs. All analyses were conducted in R 3.4.2 [38] using *α* = 0.05, and all R codes can be found in Supplemental File B.

We Jaccard transformed the relative abundance data due to our interest in understanding the presence of functions rather than abundance. We then used the package *betapart* [39] to sort beta diversity into the three components: total beta diversity (Sørensen dissimilarity: SOR), turnover (Simpson dissimilarity: SIM), and nestedness (SNE). To understand how season impacted the taxonomic structure of bat cutaneous microbial assemblages, we compared beta diversity measured as multivariate dispersion across seasons using the *betapart* function (package *vegan*; [40]) for all three metrics (SOR, SIM, and SNE). This analysis was repeated to determine if there was a difference in beta diversity across winters/years. We used permutational multivariate analysis of variance (PERMANOVA) with 999 permutations using the *adonis2* function (package *vegan*) on SOR, SIM, and SNE dissimilarity metrics to assess the influence of season and winters/years on average assemblage similarity. Previous work has shown that PERMANOVA is robust to unbalanced designs, given there is no difference in the heterogeneity of dispersions [41].

To make functional predictions based on the 16S rRNA amplicon data, we used Tax4Fun2 [42]. Tax4Fun2 uses previously annotated bacterial genomes to make functional predictions, and since many bacterial taxa lack previously sequenced genomic data, Tax4Fun2 provides a metric that shows the fraction of taxonomic units (FTU) that were unused by the program. We used a GLM to compare the FTU values between samples taken in the summer and the winter to ensure that there was no difference in how well-represented seasonal microbial assemblages were in the database.

To determine if there are seasonal shifts in putative functional assemblage structure, we followed the methods outlined above on the Tax4Fun2 predicted functions and pathways. Tax4Fun2 relies on the KEGG database for functional annotation and provides outputs for KEGG orthologs (KOs). Specifically, we generated three distance matrices for total beta diversity (SOR), turnover (SIM), and nestedness (SNE) components of Sørensen diversity using the *betapart* package [39] for both all putative functions, as well as four pathways of hypothesized importance. We selected the pathways for the biosynthesis of secondary metabolites, membrane transport, and the metabolism of terpenoids and polyketides as these have been hypothesized to play a role in host defense of fungal pathogens [21, 23]. We also selected the pathways that play a role in energy metabolism (defined in the KEGG pathway database), specifically methane metabolism, glycolysis, oxidative phosphorylation, and sulfur metabolism as these may reflect changes in bacterial ecology across seasons. We then used the *betadisper* function (package *vegan*;[40]) to compare beta diversity measured as multivariate dispersion of functional profiles across seasons and years. In order to determine if the average functional assemblage varied across seasons and years, we used a permutational multivariate analysis of variance (PERMANOVA) with 999 permutations (function *adonis2*; package *vegan*) on all three distance matrices. Additionally, to determine if there are seasonal shifts in the relative abundance of these genes within selected functional pathways, we used a Kruskal–Wallis test or a *t*-test when the assumptions were met to compare the relative abundance of genes (KOs) across seasons and years for each of the pathways.

## Results

### Effect of Season on Taxonomic Structure

**Fig. 1:**
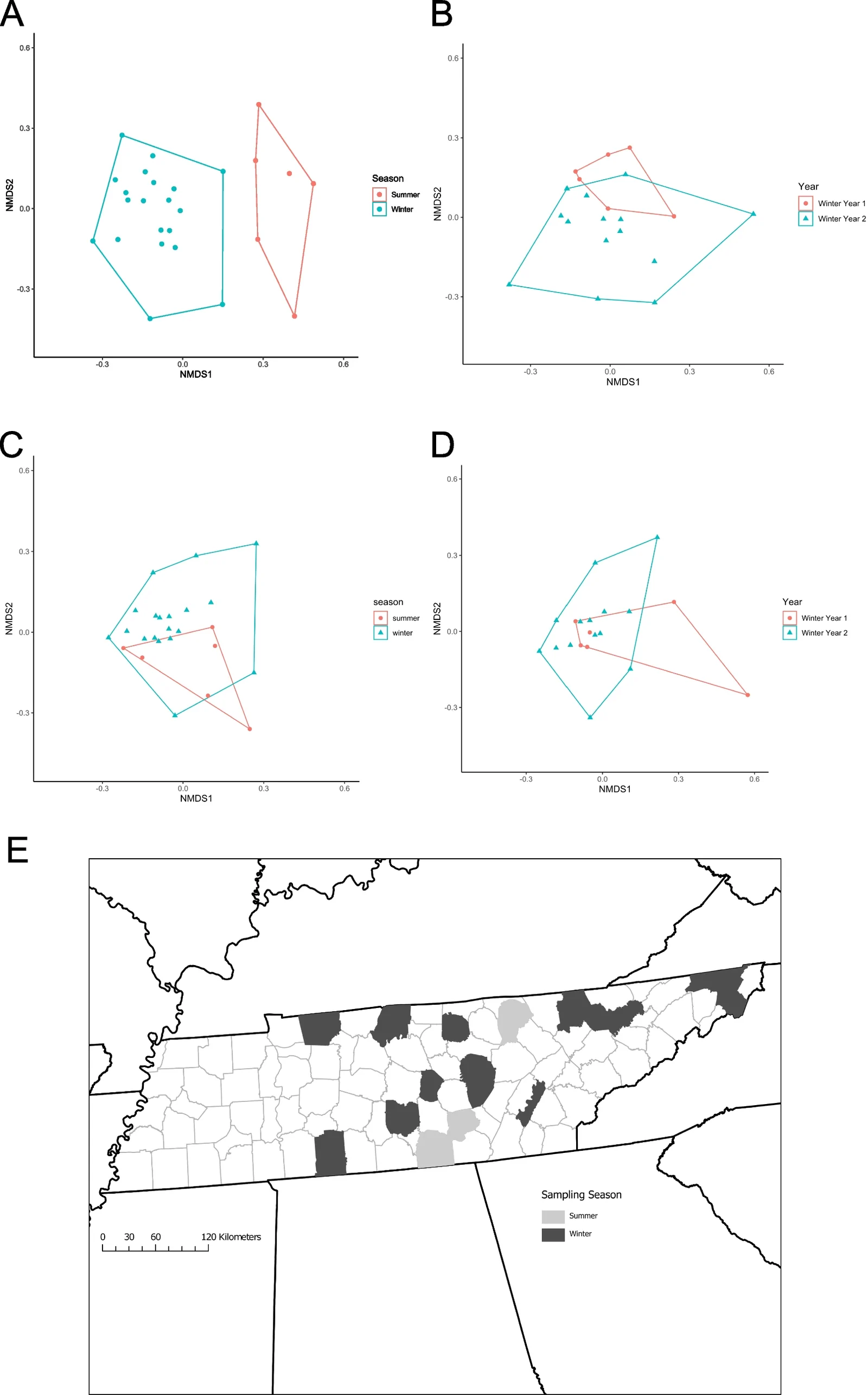
Non-metric multidimensional scaling ordination (NMDS) comparing taxonomic beta diversity of bat associated microbial assemblages across (**A**) season (stress = 0.12) and (**B**) winters (stress = 0.10). There is a significant difference in average assemblage structure across seasons (**A**; *p* < 0.05) but not across years (**B**; *p* > 0.05). (**C**) NMDS comparing functional beta diversity of bat associated microbial assemblages across season (stress = 0.06) and (**D**) winters (stress = 0.13). There is no significant difference in average functional assemblage structure across seasons (*p* > 0.05) and years (*p* > 0.05). (**E**) Distribution of sample locations, lighter-shaded counties were sampled in summer; darker counties were sampled in winter

Taxonomic beta diversity, measured as multivariate dispersion, was not significantly different across seasons (winter to summer) for any of the components of beta diversity (betadisper; SOR: *F*_1, 24_ = 0.3344, *p* = 0.582; SIM: *F*_1, 24_ = 0.0588, *p* = 0.804; SNE: *F*_1, 24_ = 2.433, *p* = 0.151; Supplemental Table C2). However, PERMANOVA indicates that the average taxonomic assemblage structure differed between bats sampled in the summer versus the winter for both total beta diversity (SOR) and for turnover (SIM; PERMANOVA; SOR: *F*_1, 25_ = 1.8388, *p* = 0.001; SIM: *F*_1, 25_ = 1.823, *p* = 0.001, Fig. 1A; Supplemental Table C3) but not for the nestedness component (PERMANOVA; SNE: *F*_1, 25_ = 1.3547, *p* = 0.377).

### Effect of Year on Taxonomic Structure

Multivariate dispersion, representing taxonomic beta diversity, was not significantly different across years (Winter 2016–2017 vs Winter 2017–2018) for any of the components of beta diversity (betadisper; SOR: *F*_1, 18_ = 2.1021, *p* = 0.153; SIM: *F*_1, 18_ = 3.4448, *p* = 0.083; SNE: *F*_1, 18_ = 0.8093, *p* = 0.378; Supplemental Table C3). Additionally, PERAMNOVA showed no significant differences in the average assemblage structure across years (PERMANOVA; SOR: *F*_1, 19_ = 1.0453, *p* = 0.26; SIM: *F*_1, 19_ = 1.1225, *p* = 0.187; SNE: *F*_1, 19_ = 0.6189, *p* = 0.529, Fig. 1B; Supplemental Table C5).

### Effect of Season on Function

On average, the percentage of OTUs used by Tax4Fun2 in forming predictions of KEGG orthologs present was 28% (average FTU = 0.72, min = 0.32, max = 0.89). The percentage of OTUs used did not significantly differ between winter and summer samples (GLM; *z* = 0.653, *p* = 0.652; Supplemental Table C6).

At the assemblage scale, across seasons, we found no significant difference in the multivariate dispersion of putative functions determined by Tax4Fun2 across all three components of beta diversity (betadisper; SOR: *F*_1, 24_ = 0.0445, *p* = 0.839; SIM: *F*_1, 24_ = 0.0138, *p* = 0.912; SNE: *F*_1, 24_ = 0.4598, *p* = 0.498; Supplemental Table C2). Additionally, PERAMANOVA revealed that there are no significant differences between the average functional KO assemblage structure between summer and winter collected samples (PERMANOVA; SOR: *F*_1, 25_ = 1.0115, *p* = 0.359; SIM: *F*_1, 25_ = 2.1803, *p* = 0.166; SNE: *F*_1, 25_ =  − 0.0116, *p* = 0.926, Fig. 1C; Supplemental Table C7).

### Effect of Year on Functional Structure

Multivariate dispersion, representing functional beta diversity, was not significantly different across years (Winter 2016–2017 vs Winter 2017–2018) for any of the components of beta diversity (betadisper; SOR: *F*_1, 18_ = 0.5913, *p* = 0.435; SIM: *F*_1, 18_ = 0.116, *p* = 0.732; SNE: *F*_1, 18_ = 0.4005, *p* = 0.564; Supplemental Table C4). Additionally, PERAMNOVA showed no significant differences in the average assemblage structure across years (PERMANOVA; SOR: *F*_1, 19_ = 1.269, *p* = 0.21; SIM: *F*_1, 19_ = 2.7607, *p* = 0.087; SNE: *F*_1, 19_ =  − 0.0264, *p* = 0.933, Fig. 1D; Supplemental Table C8).

### Fine Scale Effect of Season on Microbiome Function

**Fig. 2:**
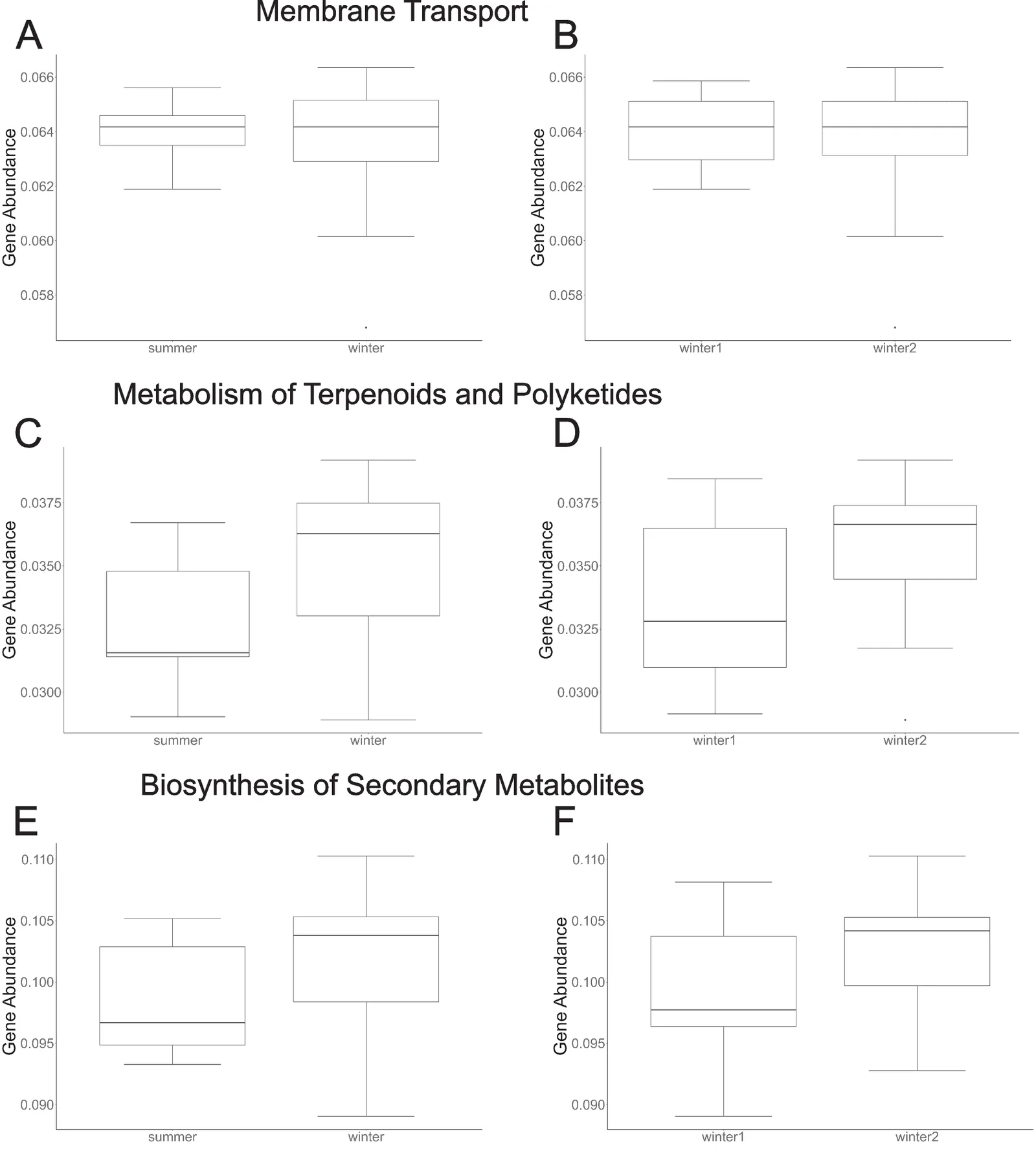
Comparison of relative abundance of genes within the pathways for protective functions (**A**) membrane transport across seasons, (**B**) membrane transport across years; (**C**) metabolism of terpenoids and polyketides across season, (**D**) metabolism of terpenoids and polyketides across years, (**E**) biosynthesis of secondary metabolites across seasons, and (**F**) biosynthesis of secondary metabolites across years. There were no significant differences in gene relative abundances across seasons or years

For genes associated with pathways of hypothesized importance in host defense (biosynthesis of secondary metabolites, membrane transport, and the metabolism of terpenoids and polyketides), there were no significant differences in beta diversity measured as multivariate dispersion (Supplemental Table C9), average assemblage structure (Supplemental Table C10-C23), or differences in gene relative abundance (Fig. 2; Supplemental Table C24) across seasons or years for any of the components of beta diversity (SOR, SIM, SNE) of each pathway.

### Fine Scale Effect of Season on Metabolic Functions

**Fig. 3:**
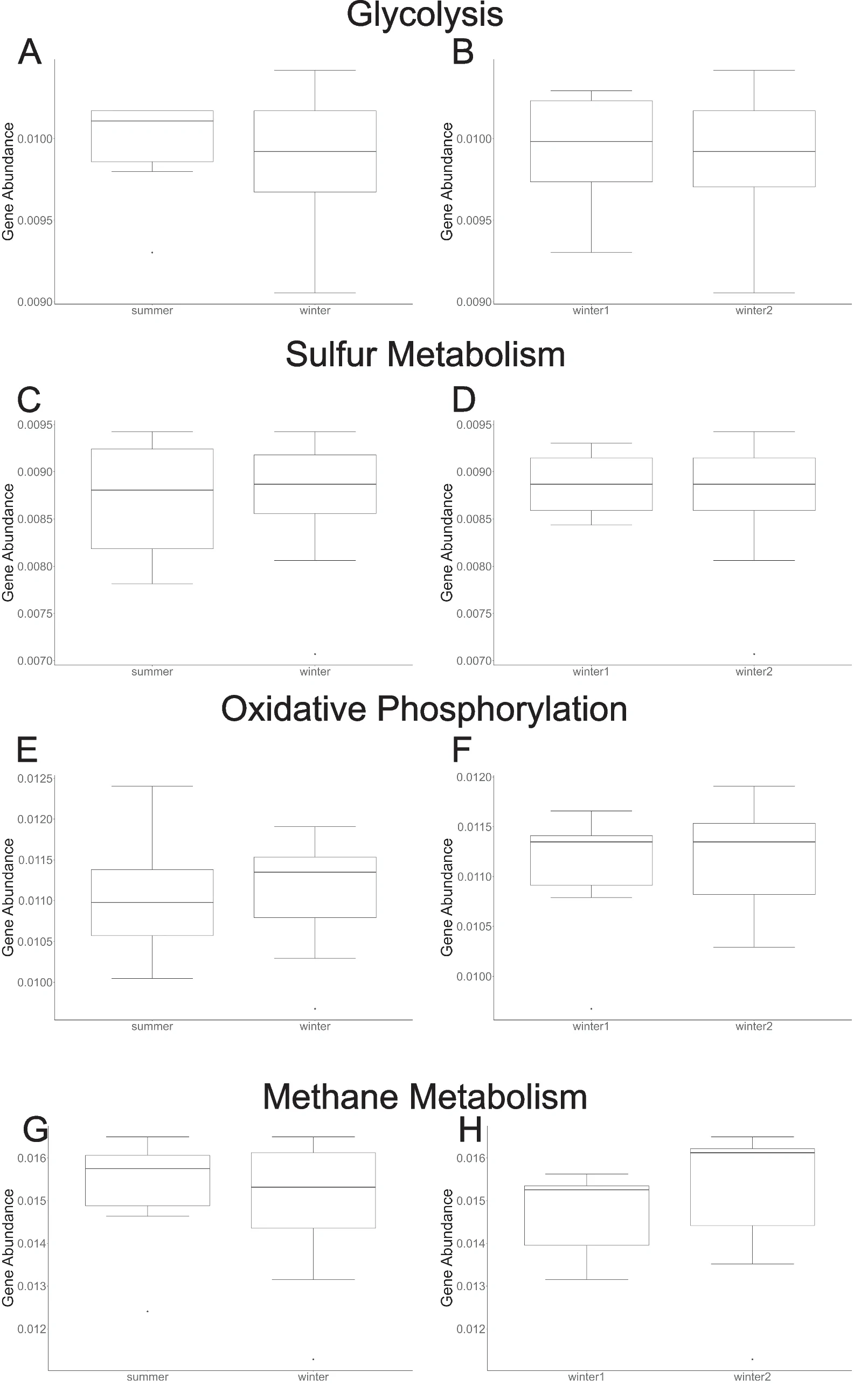
Comparison of relative abundance of genes within the pathways for metabolic functions (**A**) glycolysis across seasons, (**B**) glycolysis across years, (**C**) sulfur metabolism across seasons, (**D**) sulfur metabolism across years, (**E**) oxidative phosphorylation across seasons, (**F**) oxidative phosphorylation across years, (**G**) methane metabolism across seasons, (**H**) methane metabolism across years. There were no statistically significant differences in gene abundance across seasons or years

For genes associated with metabolic pathways (methane metabolism, glycolysis, oxidative phosphorylation, and sulfur metabolism), there were no significant differences in beta diversity measured as multivariate dispersion (Supplemental Table C11), average assemblage structure (Supplemental Tables C9–C22), or differences in gene relative abundance (Fig. 3; Supplemental Table C24) across seasons or years for any of the components of beta diversity (SOR, SIM, SNE) of each pathway.

## Discussion

Overall, our results are consistent with patterns of functional redundancy despite taxonomic variability. We found that the bat cutaneous microbial taxonomic assemblages are seasonally dynamic but functionally redundant. Specifically, bats sampled in the winter have a unique taxonomic microbial assemblage from bats sampled in the summer, but these unique assemblages have the same overall function. Interestingly when the overall taxonomic structure is compared across winters, we do not see a significant difference in structure. Additionally, patterns of functional redundancy were also observed at a finer scale, with both the structure and the abundances of genes within select functional pathways not differing across seasons or winters. Our results support previous suggestions that bat microbiomes are assembled via species-sorting mechanisms across functional groups [23]; however, our results suggest that the taxonomic structure does not necessarily vary stochastically as is observed by the conserved taxonomic structure across winters.

At the broadest scale (community level), we see a shift in taxonomic structure across seasons but a shared functional assemblage structure. Overall, there were only 306 OTUs shared across seasons, but the majority of these are found on < 50% of bats. Specifically, OTU 106 (Genus Gaiella) and OTU 103 (Genus Mycobacterium) were the only OTUs found on > 50% of bats within each season, further exemplifying the almost complete turnover in taxonomic structure across seasons. Previous work has suggested that disturbances such as pathogen presence can alter the taxonomic structure of the cutaneous microbial assemblage of bats [12, 13]. However, few studies have looked at how host-mediated disturbances, such as seasonal variation in host habitat, impact the structure of the cutaneous microbial assemblage (but see [43]). The observed shift in taxonomic structure could be due to several changes that occur at multiple scales. For example, at the landscape level, previous work has suggested that the host environment can influence the assembly of the bat cutaneous microbiome [8, 9], potentially through species sorting mechanisms [10]. Within this study, sampling was not balanced geographically, with winter samples being collected from a wider range of ecoregions (Fig. 1E). This could suggest that geographic location could be a driver of the significant difference across seasons. However, previous work on the influence of ecoregion on Tennessee bat microbial assemblages has shown that ecoregion influenced beta diversity rather than average assemblage structure [8–10]. Here, we found no significant difference in the beta diversity across seasons but did detect a difference in average assemblage structure, indicating that the difference between seasons is greater than the difference within seasons potentially attributed to environmental factors. Unfortunately, due to the structure of the data, a finer-scale assessment of the interaction between the host environment and the season was not possible. At the host level, it has been shown that the skin factors of bats, such as skin pH, vary seasonally [44]. While there is currently a lack of understanding of the interaction between host skin chemistry and cutaneous microbial assemblage structure, previous work with environmental microbial assemblages has shown that pH is a major abiotic factor influencing the structure of microbial communities [45, 46]. Interestingly, the winter bat cutaneous microbial assemblage did not vary taxonomically by year, suggesting that there is selection on the taxonomic microbial assemblage, despite previous hypotheses suggesting that taxonomic structure varies stochastically [23]. However, the results here do not necessarily conflict with the previously suggested lottery hypothesis, where assemblages are colonized randomly from a species pool of functionally equivalent species [17, 18]. Unfortunately, due to data structure and the decline of bats across Tennessee, a deeper look at how species pools vary across seasons was not possible within the current study.

Across seasons, we saw no difference in the broadscale bat cutaneous functional assemblage structure. This supports previous work suggesting microbial assemblages are maintained through species sorting mechanisms based on function rather than species identity [18]. However, this pattern could also be a result of scale, with the functions present at this level being functions that include essential processes for bacterial survival [23]. This pattern is observed across host phyla, with other studies finding essential functional pathways such as amino acid and carbohydrate metabolism being the most abundant pathways in frog microbial assemblages [21]. However, on a finer scale, across all the KEGG pathways of hypothesized importance for pathogen defense, as well as bacterial metabolism, we saw no significant differences in the relative abundance of genes across seasons or years and no differences in assemblage composition. This further supports the hypothesized functional redundancy despite taxonomic variability. Alternatively, it is possible that this observed pattern of functional redundancy is the result of functional resilience, with shifts in assemblage function decreasing in magnitude over time [15]. Assemblages exposed to repeated disturbances are often hypothesized to be more resilient to these disturbances, while novel disturbances are more likely to cause shifts in assemblage structure [15, 47]. Supporting this, previous work has shown that genes within pathways for the metabolism of terpenoids and polyketides, biosynthesis of secondary metabolites, and membrane transport are more abundant in bats that have been infected with *P. destructans* compared to *P. destructans* negative bats [23]. Taken together, these results might suggest that the bat cutaneous microbial assemblage is functionally resilient to repeated disturbances; however, further work is needed to better elucidate the functional responses of skin assemblages to repeated and novel disturbances.

It is important to note that the presence of genes and pathways as predicted by Tax4Fun2 does not necessarily indicate functional expression within the community. While Tax4Fun2 does not show what traits are being expressed at a given time, it does demonstrate the functional capacity of an assemblage. This is an indirect route and proxy for prediction of function within microbial assemblages and therefore is limited by bacterial database completeness. However, previous work has shown that for bat skin microbial assemblages, the Tax4Fun2 predicted functions are correlated with shotgun metagenomic generated results [23]. Additionally, it is important to note that on average, the functions determined by Tax4Fun2 were based on ~ 28% of OTUs per sample. We acknowledge that this is a relatively small percent of OTUs; however, this number is on par with previous work using both modeling (Tax4Fun2) as well as shotgun metagenomic sequencing [13, 20, 21, 23] and represents a general lack of understanding of the function in bacterial communities.

The objective of this study was to understand how seasonal shifts in host habitat impact the bat cutaneous microbial assemblage both taxonomically and functionally. Overall, we saw that the taxonomic groups of bat cutaneous microbial assemblages are seasonally dynamic but functionally redundant. Interestingly, despite the taxonomic shift across seasons, we found that across years, the average winter bat cutaneous microbial assemblage did not vary, suggesting selection acting on taxonomy, as well as function. These patterns were also observed at a finer scale, with no significant difference in pathways of hypothesized importance across seasons or years. Taken together, these results add to the literature and support the hypothesis of functional redundancy via assemblage-level species sorting mechanisms.

## Supplementary Information

Below is the link to the electronic supplementary material.

Supplementary file1 (DOCX 345 KB)

## Data Availability

All sequence data were submitted to GenBank SRA under the accession number PRJNA 1186968. All mothur code and all R code has been made publicly accessible in the supplemental file.
