# Supplementary material for: Bat Cutaneous Microbial Assemblage Functional Redundancy Across a Host-Mediated Disturbance
Source: Microb Ecol. 2024 Dec 21;87(1):161. doi: 10.1007/s00248-024-02480-2 (PMC11663193; doi:10.1007/s00248-024-02480-2)
Supplement: Supplementary file 1 — Supplementary file1 (DOCX 345 KB) [file 248_2024_2480_MOESM1_ESM.docx]

**Title: Bat Cutaneous Microbial Assemblage Functional Redundancy Across a Host Mediated Disturbance**

Matthew Grisnik^1^, Donald M Walker^1^

^1^ Middle Tennessee State University, Department of Biology, Murfreesboro, Tennessee 37132, USA

Correspondence Matthew Grisnik, Coastal Carolina University, Department of Biology, Conway, South Carolina 29528, USA email: mgrisnik@coastal.edu

Table of Contents

Supplemental File A mothur code 2

Supplemental File B R code 4

Supplemental File C Tables of Results 44

Supplemental File A mothur code

Mothur commands

Linux version

Using ReadLine

mothur v.1.42.1

Last updated: 01/09/2020

by

Patrick D. Schloss

Department of Microbiology & Immunology

University of Michigan

http://www.mothur.org

When using, please cite:

Schloss, P.D., et al., Introducing mothur: Open-source, platform-independent, community-supported software for describing and comparing microbial communities. Appl Environ Microbiol, 2009. 75(23):7537-41.

Distributed under the GNU General Public License

Type 'help()' for information on the commands that are available

For questions and analysis support, please visit our forum at https://forum.mothur.org

Type 'quit()' to exit program

[NOTE]: Setting random seed to 19760620.

Interactive Mode

mothur > make.contigs(file=200109_stability.txt, processors=30)

mothur > pcr.seqs(fasta=current, group=current, oligos=oligos.txt, pdiffs=1, rdoiffs=3)

mothur > pcr.seqs(fasta=current, group=current, oligos=oligos.txt, pdiffs=1, rdiffs=3)

mothur > summary.seqs(fasta=current)

mothur > screen.seqs(fasta=current, group=current, maxambig=0, maxlength=256, maxhomop=8, minlength=248)

mothur > summary.seqs(fasta=current)

mothur > unique.seqs(fasta=current)

mothur > summary.seqs(fasta=current)

mothur > count.seqs(name=current, group=current)

mothur > summary.seqs(count=current)

mothur > pcr.seqs(fasta=silva.bacteria.fasta, start=11894, end=25319, keepdots=F)

mothur > system(mv silva.bacteria.pcr.fasta silva.v4.fast)

mothur > system(mv silva.bacteria.pcr.fasta silva.v4.fasta)

mothur > system(mv silva.v4.fast silva.v4.fasta)

mothur > align.seqs(fasta=200109_stability.trim.contigs.pcr.good.unique.fasta, reference=silva.v4.fasta)

mothur > summary.seqs(fasta=current, count=current)

mothur > screen.seqs(fasta=current, count=current, start=1968, end=11550)

mothur > summary.seqs(fasta=current, count=current)

mothur > filter.seqs(fasta=current, vertical=T, trump=.)

mothur > summary.seqs(fasta=current, count=current)

mothur > unique.seqs(fasta=current, count=current)

mothur > set.current(count=200109_stability.trim.contigs.pcr.good.good.count_table, fasta=200109_stability.trim.contigs.pcr.good.unique.good.filter.fasta, processors=24)

mothur > unique.seqs(fasta=current, count=current)

mothur > summary.seqs(fasta=current, count=current)

mothur > pre.cluster(fasta=current, count=current, diffs=2)

mothur > summary.seqs(fasta=current, count=current)

mothur > chimera.uchime(fast=current, count=current, dereplicate=t)

mothur > chimera.uchime(fasta=current, count=current, dereplicate=t)

mothur > summary.seqs(count=current)

mothur > remove.seqs(fasta=current, accnos=current)

mothur > summary.seqs(fasta=current, count=current)

mothur > classify.seqs(fasta=current, count=current, reference=silva.nr_v132.pcr.align, taxonomy=silva.nr_v132.tax, cutoff=80)

mothur > summary.seqs(fasta=current, count=current)

mothur > remove.lineage(fasta=current, count=current, taxonomy=current, taxon=Chloroplast-Mitochondria-unknown-Archaea-Eukaryota)

mothur > summary.tax(taxonomy=current, count=current)

mothur > cluster.split(fasta=current, count=current, taxonomy=current, splitmethod=classify, taxlevel=4, cutoff=0.03)

mothur > summary.seqs(fasta=current, count=current)

mothur > make.shared(list=current, count=current, label=0.03)

mothur > get.groups(shared=current, groups=Negativelib1-Negativeplate9-Negativeplate8-Negativeplate6-Negativeplate5-Negativeplate4-Negativelib2-Negativelib3-Negativelib4-Negativelib5-Negativelib6)

mothur > remove.rare(shared=current, nseqs=5)

mothur > classify.otu(list=current, count=current, taxonomy=current, label=0.03)

mothur > count.groups(shared=current)

mothur > sub.sample(shared=current, size=1200)

Supplemental File B R Code

library(vegan)

library(betapart)

library(rcompanion)

library(ggplot2)

library(dplyr)

library(dplyr)

library(Tax4Fun2)

stability<-read.table(file= "stability", header=TRUE)

metadata<-read.csv(file="metadata4_2.csv")

sitemeta<-read.csv(file="sites.csv")

metadata<-merge(metadata,sitemeta, by="site")

data<-merge(metadata,stability, by="Group")

rownames(data)<-data$Group

set.seed(1138)

#########

#Taxonomic data season

######

EPFU<-subset(data, sp=="EPFU")

rownames(EPFU)<-EPFU$Group

EPFUmeta<-EPFU[1:17]

EPFUotus<-EPFU[20:23867]

rm(data, metadata, stability)

colSums(EPFUotus)

i<-(colSums(EPFUotus, na.rm=T)!=0)

EPFUotusnonzero<-EPFUotus[,i]

EPFUotuzeros<-EPFUotus[,!i]

colSums(EPFUotuzeros)

EPFUoturemovezero<-EPFUotusnonzero

colSums(EPFUoturemovezero)

i<-(colSums(EPFUoturemovezero, na.rm=T)!=1)

EPFUotusnonzero<-EPFUoturemovezero[,i]

EPFUotuzeros<-EPFUoturemovezero[,!i]

colSums(EPFUotuzeros)

EPFUoturemoveone<-EPFUotusnonzero

s<-colSums(EPFUoturemoveone)

summary(s)

PA_EPFU<-decostand(x=EPFUoturemoveone, method="pa")

betapart.core_EPFU<-betapart.core(PA_EPFU)

pair_EPFU<-beta.pair(betapart.core_EPFU, index.family="sorensen")

SOR_EPFU<-(pair_EPFU$beta.sor)

SIM_EPFU<-(pair_EPFU$beta.sim)

SNE_EPFU<-(pair_EPFU$beta.sne)

season<-EPFU$season

betadisper_SOR<-betadisper(SOR_EPFU, season, type= c("centroid"), bias.adjust=FALSE, add=FALSE)

plot(betadisper_SOR, axes=c(1,2), cex= 0.7, col = NULL, hull = TRUE, ellipse = FALSE, segments = TRUE, seg.col = "grey", label = FALSE, label.cex = 1, main= "")

(permutest.betadisper<-permutest(betadisper_SOR, pairwise=FALSE, permutations=999, parallel=getOption("mc.cores"),))

boxplot(betadisper_SOR)

#non Sig

betadisper_SIM<-betadisper(SIM_EPFU, season, type= c("centroid"), bias.adjust=FALSE, add=FALSE)

plot(betadisper_SIM, axes=c(1,2), cex= 0.7, col = NULL, hull = TRUE, ellipse = FALSE, segments = TRUE, seg.col = "grey", label = TRUE, label.cex = 1, main= "")

(permutest.betadisper<-permutest(betadisper_SIM, pairwise=FALSE, permutations=999, parallel=getOption("mc.cores"),))

boxplot(betadisper_SIM)

# non Sig

betadisper_SNE<-betadisper(SNE_EPFU, season, type= c("centroid"), bias.adjust=FALSE, add=FALSE)

plot(betadisper_SNE, axes=c(1,2), cex= 0.7, col = NULL, hull = TRUE, ellipse = FALSE, segments = TRUE, seg.col = "grey", label = TRUE, label.cex = 1, main= "")

(permutest.betadisper<-permutest(betadisper_SNE, pairwise=FALSE, permutations=999, parallel=getOption("mc.cores"),))

boxplot(betadisper_SNE)

# non Sig

adonis2(SOR_EPFU~season , permutations=999, method='bray')#sig

adonis2(SIM_EPFU~season , permutations=999, method='bray')#sig

adonis2(SNE_EPFU~season , permutations=999, method='bray')#non

skin.nmds<-metaMDS(EPFUoturemoveone, distance = "bray", k=4, try=20, trymax=2000)

data.scores <- as.data.frame(scores(skin.nmds))

data.scores

data.scores$season<- rownames(data.scores)

data.scores$season<-season

data.scores$site<-site

data.scores$county <- county

head(data.scores)

tail(data.scores)

species.scores <- as.data.frame(scores(skin.nmds, "species"))

species.scores$species <- rownames(species.scores)

head(species.scores)

Summerhull<-data.scores[data.scores$season=="summer", ][chull(data.scores[data.scores$season=="summer", c("NMDS1", "NMDS2")]), ]

winterhull<-data.scores[data.scores$season=="winter", ][chull(data.scores[data.scores$season=="winter", c("NMDS1", "NMDS2")]), ]

hull<-rbind(Summerhull, winterhull)

hull

svg("Fig1a.svg")

f1<-ggplot(data=species.scores, aes(x=NMDS1, y=NMDS2))+

geom_polygon(data=hull, aes(x=NMDS1, y=NMDS2, colour=season, group=season), fill=NA)+

geom_point(data=data.scores, aes(x=NMDS1, y=NMDS2, colour=season , shape=), size=2)+

scale_color_discrete(name="Season", labels=c("Summer", "Winter"))+

scale_shape_discrete(name="Season", labels=c("Summer", "Winter"))+

coord_fixed()+

xlim(-0.5,0.6)+

ylim(-0.5,0.6)+

theme_classic()

f1

dev.off()

rm(betadisper_SIM, betadisper_SNE, betadisper_SOR,

betapart.core_EPFU, EPFUotusnonzero, EPFUotuzeros, PA_EPFU, pair_EPFU,

permutest.betadisper, SIM_EPFU, SNE_EPFU,SOR_EPFU, season, i, s)

######

#Taxonomy Year

######

winter<-subset(EPFU, season=="winter")

wintermeta<-winter[1:17]

winterotus<-winter[20:23867]

colSums(winterotus)

i<-(colSums(winterotus, na.rm=T)!=0)

winterotusnonzero<-winterotus[,i]

winterotuzeros<-winterotus[,!i]

colSums(winterotuzeros)

winteroturemovezero<-winterotusnonzero

colSums(winteroturemovezero)

i<-(colSums(winteroturemovezero, na.rm=T)!=1)

winterotusnonzero<-winteroturemovezero[,i]

winterotuzeros<-winteroturemovezero[,!i]

colSums(winterotuzeros)

winteroturemoveone<-winterotusnonzero

s<-colSums(winteroturemoveone)

summary(s)

PA_winter<-decostand(x=winteroturemoveone, method="pa")

betapart.core_EPFUwinter<-betapart.core(PA_winter)

pair_EPFUwinter<-beta.pair(betapart.core_EPFUwinter, index.family="sorensen")

winterSOR_EPFU<-(pair_EPFUwinter$beta.sor)

winterSIM_EPFU<-(pair_EPFUwinter$beta.sim)

winterSNE_EPFU<-(pair_EPFUwinter$beta.sne)

year<-winter$year

betadisper_SORwinter<-betadisper(winterSOR_EPFU, year, type= c("centroid"), bias.adjust=FALSE, add=FALSE)

plot(betadisper_SORwinter, axes=c(1,2), cex= 0.7, col = NULL, hull = TRUE, ellipse = FALSE, segments = TRUE, seg.col = "grey", label = TRUE, label.cex = 1, main= "")

(permutest.betadisper<-permutest(betadisper_SORwinter, pairwise=FALSE, permutations=999, parallel=getOption("mc.cores"),))

boxplot(betadisper_SORwinter)

#nonSig

betadisper_SIMwinter<-betadisper(winterSIM_EPFU, year, type= c("centroid"), bias.adjust=FALSE, add=FALSE)

plot(betadisper_SIMwinter, axes=c(1,2), cex= 0.7, col = NULL, hull = TRUE, ellipse = FALSE, segments = TRUE, seg.col = "grey", label = TRUE, label.cex = 1, main= "")

(permutest.betadisper<-permutest(betadisper_SIMwinter, pairwise=FALSE, permutations=999, parallel=getOption("mc.cores"),))

boxplot(betadisper_SIMwinter)

#nonSig

betadisper_SNEwinter<-betadisper(winterSNE_EPFU, year, type= c("centroid"), bias.adjust=FALSE, add=FALSE)

plot(betadisper_SNEwinter, axes=c(1,2), cex= 0.7, col = NULL, hull = TRUE, ellipse = FALSE, segments = TRUE, seg.col = "grey", label = TRUE, label.cex = 1, main= "")

(permutest.betadisper<-permutest(betadisper_SNEwinter, pairwise=FALSE, permutations=999, parallel=getOption("mc.cores"),))

boxplot(betadisper_SNEwinter)

#nonSig

adonis2(winterSOR_EPFU~year, permutations=999, method='bray')#nonsig

adonis2(winterSIM_EPFU~year, permutations=999, method='bray')#nonsig

adonis2(winterSNE_EPFU~year, permutations=999, method='bray')#nonsig

skin.nmds<-metaMDS(winteroturemoveone, distance = "bray", k=4, try=20, trymax=2000)

data.scores <- as.data.frame(scores(skin.nmds))

data.scores

data.scores$year<- rownames(data.scores)

data.scores$year<-year

data.scores$wintersite<-wintersite

head(data.scores)

tail(data.scores)

species.scores <- as.data.frame(scores(skin.nmds, "species"))

species.scores$species <- rownames(species.scores)

head(species.scores)

yearonehull<-data.scores[data.scores$year=="winter1", ][chull(data.scores[data.scores$year=="winter1", c("NMDS1", "NMDS2")]), ]

year2hull<-data.scores[data.scores$year=="winter2", ][chull(data.scores[data.scores$year=="winter2", c("NMDS1", "NMDS2")]), ]

hull<-rbind(yearonehull, year2hull)

hull

svg("Fig1b.svg")

f1<-ggplot(data=species.scores, aes(x=NMDS1, y=NMDS2))+

geom_polygon(data=hull, aes(x=NMDS1, y=NMDS2, colour=year, group=year), fill=NA)+

geom_point(data=data.scores, aes(x=NMDS1, y=NMDS2, colour= year, shape=year), size=2)+

scale_color_discrete(name="Year", labels=c("Winter Year 1", "Winter Year 2"))+

scale_shape_discrete(name="Year", labels=c("Winter Year 1", "Winter Year 2"))+

coord_fixed()+

xlim(-0.5,0.6)+

ylim(-0.5,0.6)+

theme_classic()

f1

dev.off()

##########################

#Tax4fun2-functional level

###########################

library(Tax4Fun2)

testReferenceData(path_to_reference_data = "Tax4Fun2_ReferenceData_v2")

assignFunction(genome_folder = "C:/Users/grisn/OneDrive/Desktop/Tax4Fun2/Bins", file_extension = "fna", path_to_reference_data = "C:/Users/grisn/OneDrive/Desktop/Tax4Fun2/Tax4Fun2_ReferenceData_v2", num_of_threads = 5, fast = TRUE)

generateUserData(path_to_reference_data = "C:/Users/grisn/OneDrive/Desktop/Tax4Fun2/Tax4Fun2_ReferenceData_v2/",

path_to_user_data = "Bins", name_of_user_data = "User_Ref1",

SSU_file_extension = "_16SrRNA.ffn", KEGG_file_extension = "_funPro.txt")

generateUserDataByClustering(path_to_reference_data = "Tax4Fun2_ReferenceData_v2",

path_to_user_data = "Bins", name_of_user_data = "User_Ref1",

SSU_file_extension = "_16SrRNA.ffn", KEGG_file_extension = "_funPro.txt",

similarity_threshold = 0.99)

runRefBlast(path_to_otus = "full.fasta", path_to_reference_data = "Tax4Fun2_ReferenceData_v2",

path_to_temp_folder = "Ref99NR_withUser2", database_mode = "Ref99NR",

use_force = T, num_threads = 6, include_user_data = T,

path_to_user_data = "Bins", name_of_user_data = "KELP2")

makeFunctionalPrediction(path_to_otu_table = "210118_indicatornegmatrix.txt", path_to_reference_data = "Tax4Fun2_ReferenceData_v2",

path_to_temp_folder = "Ref99NR_withUser2", database_mode = "Ref99NR", normalize_by_copy_number = T,

min_identity_to_reference = 0.97, normalize_pathways = F, include_user_data = T, path_to_user_data = "KELP_UserData",

name_of_user_data = "KELP2")

runRefBlast(path_to_otus = "full.fasta", path_to_reference_data = "Tax4Fun2_ReferenceData_v2", path_to_temp_folder = "Kelp_Ref99NR", database_mode = "Ref99NR", use_force = T, num_threads = 6)

makeFunctionalPrediction(path_to_otu_table = "210118_indicatornegmatrix.txt", path_to_reference_data = "Tax4Fun2_ReferenceData_v2", path_to_temp_folder = "Kelp_Ref99NR", database_mode = "Ref99NR", normalize_by_copy_number = TRUE, min_identity_to_reference = 0.97, normalize_pathways = FALSE)

########

#Functional Analysis Season

########

stability<-read.csv(file= "functional_prediction_flipped.csv", header=TRUE)

metadata<-read.csv(file="metadata4.csv")

data<-merge(metadata,stability, by="Group")

rownames(data)<-data$Group

set.seed(1138)

EPFU<-subset(data, sp=="EPFU")

EPFUmeta<-EPFU[1:14]

EPFUfunfunction<-EPFU[15:8908]

rm(stability, data, metadata)

FTU<-EPFUmeta$FTU

season_fun<-EPFU$season

summary(FTU)

boxplot(FTU~season_fun)

plotNormalHistogram(FTU[season_fun=="winter"])

plotNormalHistogram(FTU[season_fun=="summer"])

m1<-glm(FTU~season_fun, family = binomial,

data=EPFU)

op<-par(mfrow=c(1,2))

qqnorm(resid(m1))

qqline(resid(m1))

plot(residuals(m1)~fitted(m1), main="residuals vs fitted")

op<-par(mfrow=c(1,1))

summary(m1)#Non sig

EPFUfunfunctionRemove<-EPFUfunfunction[,colSums(EPFUfunfunction) > 0.000006013] # remove bottom 5%

c<-colSums(EPFUfunfunctionRemove)

summary(c)

PA_fun<-decostand(x=EPFUfunfunctionRemove, method="pa")

betapart.corefun<-betapart.core(PA_fun)

pairfun<-beta.pair(PA_fun, index.family="sor")

SOR_fun<- (pairfun$beta.sor)

SIM_fun<- (pairfun$beta.sim)

SNE_fun<- (pairfun$beta.sne)

Funbetadisper_SOR<-betadisper(SOR_fun, season_fun, type= c("centroid"), bias.adjust=FALSE, add=FALSE)

plot(Funbetadisper_SOR, axes=c(1,2), cex= 0.7, col = NULL, hull = TRUE, ellipse = FALSE, segments = TRUE, seg.col = "grey", label = TRUE, label.cex = 1, main= "")

(permutest.betadisper<-permutest(Funbetadisper_SOR, pairwise=FALSE, permutations=999, parallel=getOption("mc.cores"),))

boxplot(Funbetadisper_SOR)

#Nonsig

Funbetadisper_SIM<-betadisper(SIM_fun, season_fun, type= c("centroid"), bias.adjust=FALSE, add=FALSE)

plot(Funbetadisper_SIM, axes=c(1,2), cex= 0.7, col = NULL, hull = TRUE, ellipse = FALSE, segments = TRUE, seg.col = "grey", label = TRUE, label.cex = 1, main= "")

(permutest.betadisper<-permutest(Funbetadisper_SIM, pairwise=FALSE, permutations=999, parallel=getOption("mc.cores"),))

boxplot(Funbetadisper_SIM)

#NonSig

Funbetadisper_SNE<-betadisper(SNE_fun, season_fun, type= c("centroid"), bias.adjust=FALSE, add=FALSE)

plot(Funbetadisper_SNE, axes=c(1,2), cex= 0.7, col = NULL, hull = TRUE, ellipse = FALSE, segments = TRUE, seg.col = "grey", label = TRUE, label.cex = 1, main= "")

(permutest.betadisper<-permutest(Funbetadisper_SNE, pairwise=FALSE, permutations=999, parallel=getOption("mc.cores"),))

boxplot(Funbetadisper_SNE)

#non sig

adonis2(SOR_fun~season_fun, permutations=999, method='bray')#Nonsig

adonis2(SIM_fun~season_fun, permutations=999, method='bray')#Nonsig

adonis2(SNE_fun~season_fun, permutations=999, method='bray')#Nonsig

skin.nmds<-metaMDS(EPFUfunfunctionRemove, distance = "bray", k=4, try=20, trymax=2000)

data.scores <- as.data.frame(scores(skin.nmds))

data.scores

data.scores$season<- rownames(data.scores)

data.scores$season<-season_fun

head(data.scores)

tail(data.scores)

species.scores <- as.data.frame(scores(skin.nmds, "species"))

species.scores$species <- rownames(species.scores)

head(species.scores)

Summerhull<-data.scores[data.scores$season=="summer", ][chull(data.scores[data.scores$season=="summer", c("NMDS1", "NMDS2")]), ]

winterhull<-data.scores[data.scores$season=="winter", ][chull(data.scores[data.scores$season=="winter", c("NMDS1", "NMDS2")]), ]

hull<-rbind(Summerhull, winterhull)

hull

svg("Fig2.svg")

f1<-ggplot(data=species.scores, aes(x=NMDS1, y=NMDS2))+

geom_polygon(data=hull, aes(x=NMDS1, y=NMDS2, colour=season, group=season), fill=NA)+

geom_point(data=data.scores, aes(x=NMDS1, y=NMDS2, colour=season , shape=), size=2)+

theme_classic()

f1

dev.off()

rm(betapart.corefun, Funbetadisper_SIM, Funbetadisper_SNE, Funbetadisper_SOR,

m1, op, PA_fun, pairfun, permutest.betadisper, c, FTU, season_fun,

SIM_fun,SNE_fun, SOR_fun)

###########

#Across winters

############

EPFU<-subset(EPFU, season=="winter")

EPFUmeta<-EPFU[1:14]

EPFUtaxfunfunction<-EPFU[15:ncol(EPFU)]

EPFUTaxfunfunctionRemove<-EPFUtaxfunfunction[,colSums(EPFUtaxfunfunction) > 0.000006013] # remove bottom 5%

PA_EPFU<-decostand(x=EPFUTaxfunfunctionRemove, method="pa")

betapart.core_EPFU<-betapart.core(PA_EPFU)

pair_EPFU<-beta.pair(betapart.core_EPFU, index.family="sorensen")

SOR_EPFU<-(pair_EPFU$beta.sor)

SIM_EPFU<-(pair_EPFU$beta.sim)

SNE_EPFU<-(pair_EPFU$beta.sne)

year<-EPFU$yearseason

betadisper_SOR<-betadisper(SOR_EPFU, year, type= c("centroid"), bias.adjust=FALSE, add=FALSE)

plot(betadisper_SOR, axes=c(1,2), cex= 0.7, col = NULL, hull = TRUE, ellipse = FALSE, segments = TRUE, seg.col = "grey", label = TRUE, label.cex = 1, main= "")

(permutest.betadisper<-permutest(betadisper_SOR, pairwise=FALSE, permutations=999, parallel=getOption("mc.cores"),))

boxplot(betadisper_SOR)

#non Sig

betadisper_SIM<-betadisper(SIM_EPFU, year, type= c("centroid"), bias.adjust=FALSE, add=FALSE)

plot(betadisper_SIM, axes=c(1,2), cex= 0.7, col = NULL, hull = TRUE, ellipse = FALSE, segments = TRUE, seg.col = "grey", label = TRUE, label.cex = 1, main= "")

(permutest.betadisper<-permutest(betadisper_SIM, pairwise=FALSE, permutations=999, parallel=getOption("mc.cores"),))

boxplot(betadisper_SIM)

# non Sig

betadisper_SNE<-betadisper(SNE_EPFU, year, type= c("centroid"), bias.adjust=FALSE, add=FALSE)

plot(betadisper_SNE, axes=c(1,2), cex= 0.7, col = NULL, hull = TRUE, ellipse = FALSE, segments = TRUE, seg.col = "grey", label = TRUE, label.cex = 1, main= "")

(permutest.betadisper<-permutest(betadisper_SNE, pairwise=FALSE, permutations=999, parallel=getOption("mc.cores"),))

boxplot(betadisper_SNE)

# non sig

adonis2(SOR_EPFU~year, permutations=999, method='bray')#

adonis2(SIM_EPFU~year, permutations=999, method='bray')#

adonis2(SNE_EPFU~year, permutations=999, method='bray')#

##########

#Functional Pathways of importance

##########

######

#Membrane Transport

######

Mtransport<-EPFUfunfunctionRemove %>% select(any_of(c("K01995","K01996","K01997","K01998","K01999","K02000","K02001","K02002","K02006","K02007","K02008","K02009","K02010","K02011","K02012",”K02017","K02018","K02020","K02036","K02037","K02038","K02040","K02041","K02042","K02044","K02045","K02046","K02047","K02048","K02062", "K02063","K02064","K02065","K02066","K02067","K02071","K02072","K02073","K02193","K02194","K02195","K02196","K02424","K02471","K03523","K05031","K05032","K05033","K05641","K05642","K05643","K05644","K05645","K05646","K05647","K05648","K05649","K05650","K05651","K05652","K05653","K05654","K05655","K05656","K05657","K05658","K05659","K05660","K05661","K05662","K05664","K05665","K05666","K05667","K05668","K05669","K05670","K05671","K05672","K05673","K05674","K05675","K05676","K05677","K05678","K05679","K05680","K05681","K06074","K06159","K05682","K05683","K05684","K05685","K05772","K05773","K05776","K05813","K05814","K05815","K05816","K05845","K05846","K05847","K06073","K06161","K06726","K06857","K06858","K06861","K07091","K07122","K07323","K07335","K08711","K08712","K09688","K09689","K09690","K09691","K09692","K09693","K09694","K06160","K09695","K09696","K09697","K09808","K09810","K09811","K09812","K09813","K09814","K09815","K09816","K09817","K09969","K09970","K09971","K09972","K09996","K09997","K09998","K09999","K10000","K10001","K10002","K10003","K10004","K10005","K10006","K10007","K10008","K10009","K10010","K10013","K10014","K10015","K10016","K10017","K10018","K10019","K10020","K10021","K10022","K10023","K10024","K10025","K10036","K10037","K10038","K10039","K10040","K10041","K10094","K10107","K10108","K10109","K10110","K10111","K10112","K10117","K10118","K10119","K10188","K10189","K10190","K10191","K10192","K10193","K10194","K10195","K10196","K10197","K10198","K10199","K10200","K10201","K10202","K10227","K10228","K10229","K10232","K10233","K10234","K10235","K10236","K10237","K10238","K10240","K10241","K10242","K10439","K10440","K10441","K10537","K10538","K10539","K10540","K10541","K10542","K10543","K10544","K10545","K10546","K10547","K10548","K10549","K10550","K10551","K10552","K10553","K10554","K10555","K10556","K10557","K10558","K10559","K10560","K10561","K10562","K10820","K10823","K10824","K10829","K10830","K10831","K11004","K11050","K11051","K11069","K11070","K11071","K11072","K11073","K11074","K11075","K11076","K11077","K11078","K11079","K11080","K11081","K11082","K11083","K11084","K11085","K11601","K11602","K11603","K11604","K11605","K11606","K11607","K11631","K11632","K11704","K11705","K11706","K11707","K11708","K11709","K11710","K11720","K11950","K11951","K11952","K11953","K11954","K11955","K11956","K11957","K11958","K11959","K11960","K11961","K11962","K11963","K12292","K12368","K12369","K12370","K12371","K12372","K12536","K12541","K13409","K13889","K13890","K13891","K13892","K13893","K13894","K13895","K13896","K14698","K14699","K15495","K15496","K15497","K15551","K15552","K15553","K15554","K15555","K15556","K15557","K15558","K15576","K15577","K15578","K15579","K15580","K15581","K15582","K15583","K15584","K15585","K15586","K15587","K15598","K15599","K15600","K15628","K15770","K15771","K15772","K16012","K16013","K16014","K16199","K16200","K16201","K16202","K16783","K16784","K16785","K16786","K16787","K16905","K16906","K16907","K16915","K16916","K16917","K16918","K16919","K16920","K16921","K16956","K16957","K16958","K16959","K16960","K16961","K16962","K16963","K17062","K17063","K17073","K17074","K17076","K17077","K17202","K17203","K17204","K17205","K17206","K17207","K17208","K17209","K17210","K17213","K17214","K17215","K17234","K17235","K17236","K17237","K17238","K17239","K17240","K17241","K17242","K17243","K17244","K17245","K17246","K17311","K17312","K17313","K17314","K17315","K17316","K17317","K17318","K17319","K17320","K17321","K17322","K17323","K17324","K17325","K17326","K17327","K17328","K17329","K17330","K17331","K18104","K18216","K18217","K18230","K18231","K18232","K18233","K18887","K18888","K18889","K18890","K18891","K18892","K18893","K18894","K18895","K19079","K19080","K19083","K19084","K19226","K19227","K19228","K19229","K19230","K19309","K19310","K19340","K19341","K19349","K19350","K19971","K19972","K19973","K19975","K19976","K20344","K20386","K20459","K20460","K20461","K20490","K20491","K20492","K20494","K22921","K22922","K22923","K23055","K23056","K23057","K23058","K23059","K23060","K23061","K23062","K23063","K23064","K23125","K23163","K23181","K23182","K23183","K23184","K23185","K23186","K23187","K23188","K23227","K23228","K23508","K23509","K23510","K23511","K23512","K23513","K23535","K23536","K23537","K23545","K23546","K23547","K24821","K25286","K25287","K25819","K00882","K02744","K02745","K02746","K02747","K02750","K02753","K02757","K02759","K02760","K02761","K02765","K02768","K02769","K02770","K02771","K02773","K02774","K02775","K02777","K02779","K02781","K02782","K02783","K02784","K02786","K02788","K02791","K02793","K02794","K02795","K02796","K02798","K02800","K02804","K02806","K02810","K02812","K02813","K02814","K02815","K02819","K02821","K02822","K03475","K08483","K08484","K08485","K10984","K10985","K10986","K11183","K11192","K11194","K11195","K11196","K11200","K17464","K17465","K17466","K17467","K19506","K19507","K19508","K19509","K20108","K20112","K20113","K20114","K20118","K23993","K25814","K02452","K02453","K02454","K02455","K02456","K02457","K02458","K02459","K02460","K02461","K02462","K02464","K02465","K03070","K03071","K03072","K03073","K03074","K03075","K03076","K03106","K03110","K03116","K03117","K03118","K03194","K03195","K03196","K03197","K03198","K03199","K03200","K03201","K03202","K03203","K03204","K03205","K03210","K03217","K03219","K03221","K03222","K03223","K03224","K03225","K03226","K03227","K03228","K03229","K03230","K03425","K04056","K04057","K04058","K04059","K11003","K11016","K11017","K11028","K11891","K11892","K11903","K11904","K11906","K11907","K11912","K11913","K11915","K12257","K12340","K12341","K12342","K13301")))

Mtransport<- cbind(rownames(Mtransport), data.frame(Mtransport, row.names=NULL))

names(Mtransport)[names(Mtransport) == 'rownames(Mtransport)'] <- 'Group'

Mtransportdata<-merge(EPFUmeta, Mtransport, by="Group")

Mtransportfuns<-Mtransportdata[15:558]

transportSeason<-as.factor(Mtransportdata$season)

Mtransportfuns[Mtransportfuns > 0]<-1

betapart.core_MTransfun<-betapart.core(Mtransportfuns)

pair_funMtrans<-beta.pair(betapart.core_MTransfun, index.family="sorensen")

SOR_funMtrans<-(pair_funMtrans$beta.sor)

SIM_funMtrans<-(pair_funMtrans$beta.sim)

SNE_funMtrans<-(pair_funMtrans$beta.sne)

betadisper_SOR_funMtrans <-betadisper(SOR_funMtrans, transportSeason, type= c("centroid"), bias.adjust=FALSE, add=FALSE)

plot(betadisper_SOR_funMtrans, axes=c(1,2), cex= 0.7, col = NULL, hull = TRUE, ellipse = FALSE, segments = TRUE, seg.col = "grey", label = FALSE, label.cex = 1, main= "")

(permutest.betadisper<-permutest(betadisper_SOR_funMtrans, pairwise=FALSE, permutations=999, parallel=getOption("mc.cores"),))

boxplot(betadisper_SOR_funMtrans, main="")

#non Sig

betadisper_SIM_funMtrans <-betadisper(SIM_funMtrans, transportSeason, type= c("centroid"), bias.adjust=FALSE, add=FALSE)

plot(betadisper_SIM_funMtrans, axes=c(1,2), cex= 0.7, col = NULL, hull = TRUE, ellipse = FALSE, segments = TRUE, seg.col = "grey", label = FALSE, label.cex = 1, main= "")

(permutest.betadisper<-permutest(betadisper_SIM_funMtrans, pairwise=FALSE, permutations=999, parallel=getOption("mc.cores"),))

boxplot(betadisper_SIM_funMtrans, main="")

#non Sig

betadisper_SNE_funMtrans <-betadisper(SNE_funMtrans, transportSeason, type= c("centroid"), bias.adjust=FALSE, add=FALSE)

plot(betadisper_SNE_funMtrans, axes=c(1,2), cex= 0.7, col = NULL, hull = TRUE, ellipse = FALSE, segments = TRUE, seg.col = "grey", label = FALSE, label.cex = 1, main= "")

(permutest.betadisper<-permutest(betadisper_SNE_funMtrans, pairwise=FALSE, permutations=999, parallel=getOption("mc.cores"),))

boxplot(betadisper_SNE_funMtrans, main="")

#non Sig

adonis2(SOR_funMtrans~transportSeason, data=Mtransportdata, permutations=999, method='bray')

#non Sig

adonis2(SIM_funMtrans~transportSeason, data=Mtransportdata, permutations=999, method='bray')

#non

adonis2(SNE_funMtrans~transportSeason, data=Mtransportdata, permutations=999, method='bray')

#non

MTransportAbund<-rowSums(Mtransportfuns)

MTransProp<-(MTransportAbund/(ncol(EPFUfunfunctionRemove)))

shapiro.test(MTransProp[transportSeason=="summer"])

shapiro.test(MTransProp[transportSeason=="winter"])

shapiro.test(MTransProp)

plotNormalHistogram(MTransProp[transportSeason=="summer"])

plotNormalHistogram(MTransProp[transportSeason=="winter"])

kruskal.test(MTransProp ~ transportSeason, data = Mtransportdata)

#Non

boxplot(MTransProp~transportSeason)

p<-ggplot(Mtransportdata, aes(x=transportSeason, y=MTransProp))+

stat_boxplot(geom='errorbar', linetype=1, width=0.5)+

geom_boxplot()+

xlab("")+

ylab("Gene Abundance")+

theme_classic()+

theme(axis.text=element_text(size=30))+

theme(text=element_text(size=40))

p

ggsave("WriteUp/Figs/240828_update/MTransabund.svg", width=15, height=10)

rm(m4,p, op, SIM_funMtrans,pair_funMtrans,MTransProp, MTransportAbund, SNE_funMtrans, SOR_funMtrans, betadisper_SIM_funMtrans,betadisper_SOR_funMtrans, betadisper_SNE_funMtrans,betapart.core_MTransfun )

#####

#Membrane Transport across winters

#####

winterMtransport<-subset(Mtransportdata, season=="winter")

winterMtransportfuns<-winterMtransport[15:558]

transportSeason<-as.factor(winterMtransport$year)

winterMtransportfuns[winterMtransportfuns > 0]<-1

betapart.core_MTransfun_winter<-betapart.core(winterMtransportfuns)

pair_funMtrans_w<-beta.pair(betapart.core_MTransfun_winter, index.family="sorensen")

SOR_funMtrans_winter<-(pair_funMtrans_w$beta.sor)

SIM_funMtrans_winter<-(pair_funMtrans_w$beta.sim)

SNE_funMtrans_winter<-(pair_funMtrans_w$beta.sne)

betadisper_SOR_funwinter <-betadisper(SOR_funMtrans_winter, transportSeason, type= c("centroid"), bias.adjust=FALSE, add=FALSE)

plot(betadisper_SOR_funwinter, axes=c(1,2), cex= 0.7, col = NULL, hull = TRUE, ellipse = FALSE, segments = TRUE, seg.col = "grey", label = FALSE, label.cex = 1, main= "")

(permutest.betadisper<-permutest(betadisper_SOR_funwinter, pairwise=FALSE, permutations=999, parallel=getOption("mc.cores"),))

boxplot(betadisper_SOR_funwinter, main="")

#non Sig

betadisper_SIM_funwinter <-betadisper(SIM_funMtrans_winter, transportSeason, type= c("centroid"), bias.adjust=FALSE, add=FALSE)

plot(betadisper_SIM_funwinter, axes=c(1,2), cex= 0.7, col = NULL, hull = TRUE, ellipse = FALSE, segments = TRUE, seg.col = "grey", label = FALSE, label.cex = 1, main= "")

(permutest.betadisper<-permutest(betadisper_SIM_funwinter, pairwise=FALSE, permutations=999, parallel=getOption("mc.cores"),))

boxplot(betadisper_SIM_funwinter, main="")

#non Sig

betadisper_SNE_funwinter <-betadisper(SNE_funMtrans_winter, transportSeason, type= c("centroid"), bias.adjust=FALSE, add=FALSE)

plot(betadisper_SNE_funwinter, axes=c(1,2), cex= 0.7, col = NULL, hull = TRUE, ellipse = FALSE, segments = TRUE, seg.col = "grey", label = FALSE, label.cex = 1, main= "")

(permutest.betadisper<-permutest(betadisper_SNE_funwinter, pairwise=FALSE, permutations=999, parallel=getOption("mc.cores"),))

boxplot(betadisper_SNE_funwinter, main="")

#non Sig

adonis2(SOR_funMtrans_winter~transportSeason, data=winterMtransport, permutations=999, method='bray')

#non Sig

adonis2(SIM_funMtrans_winter~transportSeason, data=winterMtransport, permutations=999, method='bray')

#non

adonis2(SNE_funMtrans_winter~transportSeason, data=winterMtransport, permutations=999, method='bray')

#non

winterMTransportAbund<-rowSums(winterMtransportfuns)

winterMTransProp<-(winterMTransportAbund/(ncol(EPFUfunfunctionRemove)))

shapiro.test(winterMTransProp[transportSeason=="winter1"])

shapiro.test(winterMTransProp[transportSeason=="winter2"])

plotNormalHistogram(winterMTransProp[transportSeason=="winter1"])

plotNormalHistogram(winterMTransProp[transportSeason=="winter2"])

kruskal.test(winterMTransProp ~ transportSeason, data = winterMtransport)

boxplot(winterMTransProp~transportSeason)

p<-ggplot(winterMtransport, aes(x=transportSeason, y=winterMTransProp))+

stat_boxplot(geom='errorbar', linetype=1, width=0.5)+

geom_boxplot()+

xlab("")+

ylab("Gene Abundance")+

theme_classic()+

theme(axis.text=element_text(size=30))+

theme(text=element_text(size=40))

p

rm(betadisper_SIM_funwinter, betadisper_SNE_funwinter, betadisper_SOR_funwinter,

betapart.core_MTransfun_winter, Mtransport, Mtransportdata, Mtransportfuns,

pari_funMtrans, pair_funMtrans_w, permutest.betadisper, winterMtransport, winterMtransportfuns,

MtransportAbund, MtrasnProp, SIM_funMtrans_winter, SNE_funMtrans_winter,

SOR_funMtrans_winter, transportSeason, winterMTransProp,winterMTransportAbund, winterMtransProp)

#####

#MTP

#####

MTP<-EPFUfunfunctionRemove %>% select(any_of(c("K00021","K00022","K00054","K00067","K00099","K00128","K00216","K00511","K00514","K00587","K00615","K00626","K00632","K00787","K00795","K00801","K00804","K00805","K00806","K00869","K00919","K00938","K00973","K00991","K01252","K01597","K01640","K01641","K01662","K01692","K01710","K01770","K01779","K01782","K01790","K01823","K01825","K01851","K02291","K02292","K02293","K02294","K02361","K02362","K02363","K02364","K02523","K02552","K03186","K03526","K03527","K04778","K04780","K04781","K04782","K04783","K04784","K04785","K04786","K04787","K04788","K04789","K04790","K04791","K04792","K04793","K05355","K05356","K05551","K05552","K05553","K05554","K05555","K05556","K05906","K05954","K05955","K06013","K06045","K06443","K06444","K06981","K07381","K07382","K08658","K08977","K09128","K09483","K09835","K09836","K09837","K09838","K09839","K09840","K09841","K09842","K09843","K09844","K09845","K09846","K09847","K09879","K10027","K10156","K10187","K10208","K10209","K10210","K10211","K10212","K10533","K10960","K11731","K11778","K12237","K12238","K12239","K12240","K12241","K12249","K12250","K12420","K12451","K12466","K12503","K12504","K12505","K12506","K12645","K12710","K12742","K12957","K13273","K13306","K13307","K13308","K13309","K13310","K13311","K13312","K13313","K13315","K13316","K13317","K13318","K13319","K13322","K13326","K13327","K13328","K13329","K13330","K13332","K13774","K13775","K13776","K13777","K13778","K13779","K13787","K13789","K14066","K14173","K14174","K14175","K14176","K14177","K14178","K14179","K14180","K14181","K14182","K14183","K14184","K14185","K14186","K14215","K14244","K14249","K14250","K14251","K14252","K14253","K14254","K14255","K14256","K14257","K14593","K14594","K14595","K14596","K14597","K14598","K14605","K14606","K14626","K14627","K14628","K14629","K14630","K14631","K14632","K14633","K14730","K14731","K14732","K14733","K15314","K15315","K15320","K15466","K15467","K15472","K15652","K15653","K15654","K15655","K15656","K15661","K15662","K15663","K15664","K15665","K15666","K15667","K15668","K15670","K15671","K15672","K15673","K15681","K15744","K15745","K15746","K15747","K15748","K15793","K15794","K15795","K15796","K15797","K15798","K15799","K15800","K15801","K15802","K15803","K15804","K15805","K15806","K15807","K15808","K15809","K15810","K15811","K15812","K15813","K15814","K15815","K15816","K15817","K15818","K15819","K15820","K15821","K15822","K15823","K15884","K15885","K15886","K15887","K15888","K15889","K15890","K15891","K15892","K15907","K15926","K15927","K15928","K15929","K15930","K15931","K15932","K15933","K15934","K15935","K15936","K15937","K15938","K15939","K15941","K15942","K15943","K15944","K15945","K15946","K15947","K15948","K15949","K15950","K15951","K15952","K15953","K15954","K15955","K15956","K15957","K15958","K15959","K15960","K15961","K15962","K15963","K15964","K15965","K15966","K15967","K15968","K15969","K15970","K15971","K15972","K16015","K16016","K16017","K16018","K16019","K16020","K16021","K16022","K16023","K16024","K16025","K16026","K16027","K16028","K16029","K16030","K16031","K16032","K16033","K16034","K16035","K16036","K16037","K16038","K16039","K16093","K16094","K16095","K16096","K16097","K16098","K16099","K16100","K16101","K16102","K16103","K16104","K16105","K16106","K16107","K16108","K16109","K16110","K16111","K16112","K16113","K16114","K16115","K16116","K16117","K16118","K16122","K16123","K16124","K16125","K16126","K16127","K16128","K16129","K16130","K16131","K16132","K16133","K16134","K16204","K16205","K16206","K16207","K16421","K16422","K16423","K16424","K16425","K16426","K16427","K16428","K16429","K16430","K16431","K16432","K16433","K16434","K16435","K16436","K16437","K16438","K16439","K16440","K16442","K16443","K16444","K16445","K16446","K16447","K16448","K17625","K17819","K17832","K17841","K17842","K17911","K17912","K17913","K17942","K17947","K18109","K18110","K18111","K18112","K18221","K18689","K18690","K18836","K19010","K19011","K19177","K19180","K19566","K19567","K19568","K19569","K19570","K19652","K19653","K19853","K19854","K19855","K19856","K19857","K19858","K19859","K20156","K20159","K20420","K20421","K20422","K20423","K20561","K20611","K20616","K20658","K20659","K20678","K20679","K20680","K20681","K20682","K21159","K21160","K21161","K21162","K21163","K21164","K21165","K21166","K21167","K21168","K21169","K21170","K21171","K21172","K21173","K21174","K21175","K21176","K21177","K21178","K21179","K21180","K21181","K21182","K21184","K21185","K21186","K21187","K21188","K21189","K21190","K21191","K21192","K21193","K21194","K21209","K21210","K21211","K21212","K21213","K21214","K21215","K21216","K21221","K21222","K21223","K21224","K21225","K21226","K21227","K21228","K21229","K21230","K21254","K21255","K21256","K21257","K21258","K21259","K21260","K21261","K21262","K21263","K21268","K21273","K21274","K21275","K21297","K21301","K21325","K21326","K21327","K21328","K21329","K21330","K21331","K21332","K21333","K21335","K21336","K21337","K21927","K21928","K21984","K22064","K22065","K22445","K22492","K22502","K22813","K23037","K23144","K23987","K24108","K24109","K24110","K24112","K24147","K24873","K25072","K25073","K25074","K25517","K25518","K26054","K26056")))

MTP<- cbind(rownames(MTP), data.frame(MTP, row.names=NULL))

names(MTP)[names(MTP) == 'rownames(MTP)'] <- 'Group'

MTPdata<-merge(EPFUmeta, MTP, by="Group")

MTPfuns<-MTPdata[15:347]

avgfMTPseason<-as.factor(MTPdata$season)

MTPfuns[MTPfuns > 0]<-1

betapart.core_MTPfun<-betapart.core(MTPfuns)

pair_funMTP<-beta.pair(betapart.core_MTPfun, index.family="sorensen")

SOR_funMTP<-(pair_funMTP$beta.sor)

SIM_funMTP<-(pair_funMTP$beta.sim)

SNE_funMTP<-(pair_funMTP$beta.sne)

betadisper_SOR_funMTP <-betadisper(SOR_funMTP, avgfMTPseason, type= c("centroid"), bias.adjust=FALSE, add=FALSE)

plot(betadisper_SOR_funMTP, axes=c(1,2), cex= 0.7, col = NULL, hull = TRUE, ellipse = FALSE, segments = TRUE, seg.col = "grey", label = FALSE, label.cex = 1, main= "")

(permutest.betadisper<-permutest(betadisper_SOR_funMTP, pairwise=FALSE, permutations=999, parallel=getOption("mc.cores"),))

boxplot(betadisper_SOR_funMTP, main="")

#non Sig

betadisper_SIM_funMTP <-betadisper(SIM_funMTP, avgfMTPseason, type= c("centroid"), bias.adjust=FALSE, add=FALSE)

plot(betadisper_SIM_funMTP, axes=c(1,2), cex= 0.7, col = NULL, hull = TRUE, ellipse = FALSE, segments = TRUE, seg.col = "grey", label = FALSE, label.cex = 1, main= "")

(permutest.betadisper<-permutest(betadisper_SIM_funMTP, pairwise=FALSE, permutations=999, parallel=getOption("mc.cores"),))

boxplot(betadisper_SIM_funMTP, main="")

#non Sig

betadisper_SNE_funMTP <-betadisper(SNE_funMTP, avgfMTPseason, type= c("centroid"), bias.adjust=FALSE, add=FALSE)

plot(betadisper_SNE_funMTP, axes=c(1,2), cex= 0.7, col = NULL, hull = TRUE, ellipse = FALSE, segments = TRUE, seg.col = "grey", label = FALSE, label.cex = 1, main= "")

(permutest.betadisper<-permutest(betadisper_SNE_funMTP, pairwise=FALSE, permutations=999, parallel=getOption("mc.cores"),))

boxplot(betadisper_SNE_funMTP, main="")

#non Sig

adonis2(SOR_funMTP~avgfMTPseason, data=MTPdata, permutations=999, method='bray')

#non

adonis2(SIM_funMTP~avgfMTPseason, data=MTPdata, permutations=999, method='bray')

#non

adonis2(SNE_funMTP~avgfMTPseason, data=MTPdata, permutations=999, method='bray')

#non

MTransAbund<-rowSums(MTPfuns)

MTransProp<-(MTransAbund/(ncol(EPFUfunfunctionRemove)))

shapiro.test(MTransProp[avgfMTPseason=="winter"])

shapiro.test(MTransProp[avgfMTPseason=="summer"])

plotNormalHistogram(MTransProp[avgfMTPseason=="winter"])

plotNormalHistogram(MTransProp[avgfMTPseason=="summer"])

bartlett.test(MTransProp~avgfMTPseason)

t.test(MTransProp ~ avgfMTPseason, data = MTPdata)

boxplot(MTransProp~avgfMTPseason)

p<-ggplot(MTPdata, aes(x=avgfMTPseason, y=MTransProp))+

stat_boxplot(geom='errorbar', linetype=1, width=0.5)+

geom_boxplot()+

xlab("")+

ylab("Gene Abundance")+

theme_classic()+

theme(axis.text=element_text(size=30))+

theme(text=element_text(size=40))

p

ggsave("WriteUp/Figs/240828_update/MTPabund.svg", width=15, height=10)

rm(betadisper_SIM_funMTP, betadisper_SNE_funMTP, betadisper_SOR_funMTP,

betapart.core_MTPfun, m5, MTP, op, p , pair_funMTP, permutest.betadisper, MTransAbund,

MTransProp, SIM_funMTP, SNE_funMTP, SOR_funMTP, avgfMTPseason)

#####

#MTP across winters

#####

winterMtp<-subset(MTPdata, season=="winter")

winterMtpfuns<-winterMtp[15:347]

avgfMTPseason<-as.factor(winterMtp$yearseason )

winterMtpfuns[winterMtpfuns > 0]<-1

betapart.core_MTPfun_winter<-betapart.core(winterMtpfuns)

pair_funMTP_winter<-beta.pair(betapart.core_MTPfun_winter, index.family="sorensen")

SOR_funMTP_w<-(pair_funMTP_winter$beta.sor)

SIM_funMTP_w<-(pair_funMTP_winter$beta.sim)

SNE_funMTP_w<-(pair_funMTP_winter$beta.sne)

betadisper_SOR_funMTP_w <-betadisper(SOR_funMTP_w, avgfMTPseason, type= c("centroid"), bias.adjust=FALSE, add=FALSE)

plot(betadisper_SOR_funMTP_w, axes=c(1,2), cex= 0.7, col = NULL, hull = TRUE, ellipse = FALSE, segments = TRUE, seg.col = "grey", label = FALSE, label.cex = 1, main= "")

(permutest.betadisper<-permutest(betadisper_SOR_funMTP_w, pairwise=FALSE, permutations=999, parallel=getOption("mc.cores"),))

boxplot(betadisper_SOR_funMTP_w, main="")

#non Sig

betadisper_SIM_funMTP <-betadisper(SIM_funMTP_w, avgfMTPseason, type= c("centroid"), bias.adjust=FALSE, add=FALSE)

plot(betadisper_SIM_funMTP, axes=c(1,2), cex= 0.7, col = NULL, hull = TRUE, ellipse = FALSE, segments = TRUE, seg.col = "grey", label = FALSE, label.cex = 1, main= "")

(permutest.betadisper<-permutest(betadisper_SIM_funMTP, pairwise=FALSE, permutations=999, parallel=getOption("mc.cores"),))

boxplot(betadisper_SIM_funMTP, main="")

#non Sig

betadisper_SNE_funMTP <-betadisper(SNE_funMTP_w, avgfMTPseason, type= c("centroid"), bias.adjust=FALSE, add=FALSE)

plot(betadisper_SNE_funMTP, axes=c(1,2), cex= 0.7, col = NULL, hull = TRUE, ellipse = FALSE, segments = TRUE, seg.col = "grey", label = FALSE, label.cex = 1, main= "")

(permutest.betadisper<-permutest(betadisper_SNE_funMTP, pairwise=FALSE, permutations=999, parallel=getOption("mc.cores"),))

boxplot(betadisper_SNE_funMTP, main="")

#non Sig

adonis2(SOR_funMTP_w~avgfMTPseason, data=winterMtp, permutations=999, method='bray')

#non

adonis2(SIM_funMTP_w~avgfMTPseason, data=winterMtp, permutations=999, method='bray')

#non

adonis2(SNE_funMTP_w~avgfMTPseason, data=winterMtp, permutations=999, method='bray')

#non

MTransAbund_winter<-rowSums(winterMtpfuns)

MTransProp_winter<-(MTransAbund_winter/(ncol(EPFUfunfunctionRemove)))

shapiro.test(MTransProp_winter[avgfMTPseason=="winter1"])

shapiro.test(MTransProp_winter[avgfMTPseason=="winter2"])

plotNormalHistogram(MTransProp_winter[avgfMTPseason=="winter1"])

plotNormalHistogram(MTransProp_winter[avgfMTPseason=="winter2"])

bartlett.test(MTransProp_winter~avgfMTPseason)

t.test(MTransProp_winter ~ avgfMTPseason, data = winterMtp)

#non sig

boxplot(MTransAbund_winter~avgfMTPseason)

rm(betapart.core_MTPfun_winter,MTPdata, MTPfuns, res_aov, winterMTP, winterMtpfuns,

avgfMTPseason, c, winterMtp, MTransAbund_winter, season_fun, MTransProp_winter,

SIM_funMTP_w, SNE_funMTP_w, betadisper_SOR_funMTP_w,SOR_funMTP_w, pair_funMTP_winter, permutest.betadisper)

#####

#Biosynth Secondary Metabolites

#####

BSM<-EPFUfunfunctionRemove %>% select(any_of(c("K00001","K00002","K00003","K00004","K00006","K00010","K00013","K00014","K00015","K00016","K00018",

"K00021","K00022","K00024","K00025","K00026","K00030","K00031","K00033","K00036","K00049","K00052",

"K00053","K00054","K00057","K00058","K00059","K00064","K00067","K00077","K00082","K00083","K00088",

"K00090","K00096","K00099","K00104","K00105","K00106","K00111","K00112","K00113","K00114","K00115",

"K00116","K00117","K00121","K00128","K00129","K00133","K00134","K00138","K00143","K00145","K00147",

"K00149","K00150","K00161","K00162","K00163","K00164","K00166","K00167","K00169","K00170","K00171",

"K00172","K00174","K00175","K00176","K00177","K00189","K00208","K00209","K00211","K00213","K00214",

"K00215","K00216","K00218","K00220","K00222","K00223","K00224","K00225","K00227","K00228","K00230",

"K00231","K00232","K00234","K00235","K00236","K00237","K00239","K00240","K00241","K00242","K00244",

"K00245","K00246","K00247","K00248","K00249","K00252","K00263","K00264","K00265","K00266","K00270",

"K00271","K00273","K00274","K00276","K00279","K00281","K00282","K00283","K00286","K00290","K00293",

"K00307","K00318","K00355","K00382","K00422","K00430","K00435","K00454","K00475","K00487","K00491",

"K00495","K00501","K00505","K00510","K00511","K00514","K00544","K00547","K00548","K00549","K00550",

"K00551","K00559","K00568","K00570","K00587","K00588","K00589","K00591","K00600","K00601","K00602",

"K00605","K00606","K00611","K00615","K00616","K00618","K00619","K00620","K00622","K00626","K00627",

"K00629","K00631","K00632","K00640","K00641","K00643","K00645","K00651","K00655","K00658","K00660",

"K00688","K00693","K00695","K00696","K00697","K00699","K00700","K00703","K00705","K00750","K00760",

"K00764","K00765","K00766","K00769","K00787","K00789","K00791","K00793","K00794","K00795","K00800",

"K00801","K00804","K00805","K00806","K00808","K00811","K00812","K00813","K00815","K00816","K00817",

"K00818","K00819","K00821","K00825","K00826","K00827","K00830","K00831","K00832","K00835","K00838",

"K00841","K00844","K00845","K00847","K00850","K00851","K00861","K00865","K00869","K00872","K00873",

"K00886","K00891","K00895","K00901","K00918","K00919","K00927","K00928","K00930","K00931","K00938",

"K00939","K00940","K00944","K00948","K00953","K00955","K00956","K00957","K00958","K00963","K00966",

"K00971","K00973","K00975","K00981","K00991","K00993","K00998","K01004","K01006","K01007","K01047",

"K01053","K01057","K01058","K01060","K01068","K01075","K01079","K01080","K01081","K01084","K01086",

"K01087","K01089","K01091","K01092","K01114","K01115","K01176","K01177","K01187","K01188","K01193",

"K01194","K01195","K01196","K01200","K01203","K01208","K01214","K01236","K01239","K01252","K01424",

"K01434","K01438","K01476","K01478","K01490","K01492","K01496","K01497","K01498","K01509","K01510",

"K01513","K01521","K01523","K01568","K01575","K01579","K01580","K01581","K01582","K01583","K01584",

"K01585","K01586","K01587","K01588","K01589","K01590","K01592","K01593","K01596","K01597","K01599",

"K01601","K01602","K01609","K01610","K01613","K01616","K01620","K01622","K01623","K01624","K01626",

"K01637","K01638","K01640","K01641","K01647","K01648","K01649","K01652","K01653","K01655","K01656",

"K01657","K01658","K01661","K01662","K01663","K01675","K01676","K01677","K01678","K01679","K01681",

"K01682","K01687","K01689","K01692","K01693","K01694","K01695","K01696","K01697","K01698","K01702",

"K01703","K01704","K01705","K01710","K01713","K01714","K01719","K01723","K01733","K01735","K01736",

"K01738","K01739","K01742","K01749","K01750","K01752","K01754","K01755","K01756","K01757","K01758",

"K01760","K01762","K01770","K01772","K01774","K01778","K01782","K01783","K01785","K01790","K01792",

"K01803","K01807","K01808","K01809","K01810","K01814","K01817","K01823","K01824","K01825","K01834",

"K01835","K01837","K01840","K01841","K01845","K01850","K01851","K01852","K01853","K01858","K01859",

"K01885","K01895","K01899","K01900","K01902","K01903","K01904","K01911","K01913","K01914","K01918",

"K01923","K01933","K01940","K01945","K01946","K01952","K01953","K01957","K01961","K01962","K01963",

"K01964","K01965","K01966","K02078","K02160","K02203","K02204","K02257","K02259","K02291","K02292",

"K02293","K02294","K02302","K02303","K02304","K02361","K02362","K02363","K02364","K02437","K02439",

"K02446","K02492","K02495","K02496","K02500","K02501","K02502","K02523","K02548","K02549","K02551",

"K02552","K02566","K02626","K02858","K03179","K03181","K03182","K03183","K03184","K03185","K03186",

"K03334","K03340","K03366","K03378","K03403","K03404","K03405","K03428","K03526","K03527","K03621",

"K03737","K03781","K03782","K03783","K03784","K03785","K03786","K03787","K03794","K03809","K03816",

"K03823","K03841","K03856","K03894","K03895","K03896","K03897","K04022","K04034","K04035","K04036",

"K04037","K04038","K04039","K04040","K04041","K04072","K04092","K04093","K04120","K04121","K04122",

"K04123","K04124","K04125","K04126","K04127","K04128","K04339","K04340","K04341","K04342","K04486",

"K04516","K04517","K04518","K04781","K04782","K05277","K05278","K05279","K05280","K05281","K05282",

"K05342","K05343","K05349","K05350","K05353","K05354","K05355","K05356","K05357","K05358","K05359",

"K05369","K05370","K05371","K05375","K05525","K05551","K05552","K05553","K05554","K05555","K05556",

"K05597","K05602","K05821","K05822","K05823","K05824","K05825","K05828","K05829","K05830","K05887",

"K05894","K05901","K05906","K05917","K05928","K05933","K05942","K05953","K05954","K05955","K05957",

"K05992","K06001","K06013","K06044","K06045","K06125","K06126","K06127","K06134","K06208","K06209",

"K06443","K06444","K06859","K06863","K06892","K06900","K06928","K06981","K06998","K07024","K07029",

"K07094","K07145","K07215","K07226","K07381","K07382","K07384","K07385","K07404","K07405","K07409",

"K07419","K07508","K07509","K07511","K07513","K07514","K07515","K07748","K07750","K08074","K08081",

"K08099","K08100","K08101","K08233","K08241","K08242","K08243","K08246","K08248","K08249","K08289",

"K08295","K08591","K08658","K08680","K08683","K08693","K08695","K08730","K08973","K08977","K09128",

"K09459","K09460","K09478","K09483","K09587","K09588","K09589","K09590","K09591","K09699","K09753",

"K09754","K09755","K09756","K09757","K09827","K09828","K09829","K09831","K09832","K09833","K09834",

"K09835","K09836","K09837","K09838","K09839","K09840","K09841","K09842","K09843","K09844","K09845",

"K09846","K09847","K09879","K09913","K10027","K10046","K10047","K10106","K10150","K10156","K10187",

"K10203","K10205","K10206","K10208","K10209","K10210","K10211","K10212","K10226","K10244","K10245",

"K10246","K10247","K10248","K10249","K10250","K10251","K10258","K10525","K10526","K10527","K10528",

"K10529","K10533","K10566","K10703","K10705","K10717","K10757","K10760","K10775","K10814","K10815",

"K10816","K10817","K10852","K10960","K10977","K10978","K11067","K11175","K11176","K11188","K11258",

"K11262","K11263","K11333","K11334","K11335","K11336","K11337","K11358","K11381","K11410","K11472",

"K11473","K11517","K11529","K11532","K11645","K11646","K11731","K11751","K11752","K11753","K11755",

"K11778","K11782","K11783","K11784","K11785","K11787","K11788","K11808","K11812","K11813","K11818",

"K11819","K11820","K11821","K12047","K12073","K12153","K12154","K12156","K12250","K12316","K12317",

"K12338","K12355","K12356","K12406","K12407","K12420","K12447","K12451","K12466","K12467","K12501",

"K12502","K12503","K12504","K12505","K12506","K12524","K12525","K12526","K12570","K12628","K12629",

"K12630","K12631","K12632","K12633","K12634","K12635","K12636","K12637","K12638","K12639","K12640",

"K12643","K12644","K12645","K12657","K12659","K12673","K12674","K12675","K12676","K12677","K12692",

"K12693","K12694","K12695","K12696","K12697","K12698","K12699","K12701","K12702","K12703","K12704",

"K12705","K12707","K12708","K12709","K12710","K12711","K12712","K12713","K12714","K12719","K12720",

"K12721","K12722","K12723","K12724","K12729","K12730","K12731","K12742","K12743","K12744","K12745",

"K12747","K12748","K12901","K12902","K12903","K12904","K12905","K12906","K12907","K12908","K12909",

"K12910","K12911","K12912","K12913","K12914","K12915","K12917","K12918","K12919","K12920","K12921",

"K12922","K12923","K12924","K12925","K12926","K12927","K12928","K12929","K12930","K12934","K12935",

"K12936","K12939","K12957","K12972","K13027","K13029","K13030","K13031","K13032","K13033","K13034",

"K13035","K13037","K13051","K13063","K13064","K13065","K13066","K13067","K13068","K13070","K13071",

"K13077","K13079","K13081","K13082","K13083","K13222","K13223","K13224","K13225","K13226","K13227",

"K13229","K13230","K13231","K13232","K13233","K13234","K13235","K13240","K13241","K13242","K13257",

"K13258","K13259","K13260","K13261","K13262","K13263","K13265","K13266","K13267","K13269","K13272",

"K13273","K13306","K13307","K13308","K13309","K13310","K13311","K13312","K13313","K13315","K13316",

"K13317","K13318","K13319","K13320","K13322","K13326","K13327","K13328","K13329","K13330","K13332",

"K13371","K13372","K13373","K13382","K13383","K13384","K13385","K13386","K13387","K13389","K13390",

"K13391","K13392","K13393","K13394","K13395","K13396","K13397","K13398","K13400","K13401","K13427",

"K13492","K13493","K13494","K13495","K13496","K13497","K13498","K13501","K13503","K13506","K13507",

"K13508","K13509","K13513","K13517","K13519","K13523","K13542","K13543","K13544","K13545","K13546",

"K13547","K13548","K13549","K13550","K13551","K13552","K13553","K13554","K13555","K13556","K13557",

"K13559","K13560","K13561","K13562","K13563","K13564","K13565","K13574","K13600","K13601","K13602",

"K13603","K13604","K13605","K13606","K13607","K13608","K13644","K13679","K13713","K13774","K13775",

"K13776","K13777","K13778","K13779","K13787","K13789","K13799","K13810","K13811","K13821","K13829",

"K13830","K13832","K13853","K13937","K13951","K13952","K13953","K13954","K13979","K13997","K14028",

"K14029","K14036","K14037","K14038","K14039","K14040","K14041","K14042","K14043","K14044","K14045",

"K14046","K14047","K14066","K14085","K14130","K14132","K14134","K14135","K14152","K14155","K14157",

"K14163","K14170","K14173","K14174","K14176","K14177","K14178","K14179","K14180","K14182","K14183",

"K14184","K14186","K14187","K14190","K14215","K14244","K14245","K14246","K14249","K14250","K14251",

"K14252","K14253","K14254","K14255","K14256","K14257","K14260","K14266","K14271","K14272","K14329",

"K14366","K14367","K14368","K14369","K14370","K14371","K14372","K14373","K14374","K14375","K14423",

"K14424","K14454","K14455","K14577","K14593","K14594","K14595","K14596","K14597","K14598","K14605",

"K14606","K14621","K14626","K14627","K14628","K14629","K14630","K14631","K14632","K14633","K14641",

"K14642","K14652","K14656","K14674","K14677","K14681","K14682","K14730","K14731","K14732","K14733",

"K14759","K14760","K14975","K14976","K14984","K15036","K15037","K15086","K15087","K15088","K15089",

"K15090","K15091","K15092","K15093","K15094","K15095","K15096","K15097","K15098","K15099","K15226",

"K15227","K15230","K15231","K15314","K15315","K15316","K15320","K15397","K15404","K15405","K15467",

"K15472","K15506","K15633","K15634","K15635","K15639","K15652","K15728","K15741","K15742","K15744",

"K15745","K15746","K15747","K15748","K15759","K15774","K15775","K15776","K15777","K15778","K15779",

"K15780","K15791","K15793","K15800","K15808","K15812","K15813","K15814","K15815","K15816","K15817",

"K15819","K15821","K15823","K15849","K15884","K15885","K15886","K15887","K15889","K15890","K15891",

"K15892","K15893","K15907","K15916","K15918","K15919","K15926","K15927","K15928","K15929","K15930",

"K15931","K15932","K15933","K15934","K15935","K15936","K15937","K15938","K15939","K15941","K15942",

"K15943","K15944","K15945","K15946","K15947","K15948","K15949","K15950","K15951","K15952",

"K15953","K15954","K15955","K15956","K15957","K15958","K15959","K15960","K15961","K15962","K15963",

"K15964","K15965","K15966","K15967","K15968","K15969","K15970","K15971","K15972","K15988","K15989",

"K15990","K15991","K15992","K15993","K15994","K15995","K15996","K15997","K15998","K15999","K16000",

"K16001","K16002","K16003","K16004","K16005","K16006","K16007","K16008","K16009","K16010","K16011",

"K16015","K16016","K16017","K16018","K16019","K16020","K16021","K16023","K16033","K16034","K16035",

"K16036","K16037","K16038","K16039","K16040","K16055","K16082","K16083","K16084","K16085","K16086",

"K16149","K16150","K16153","K16207","K16265","K16266","K16305","K16306","K16339","K16342","K16343",

"K16368","K16369","K16370","K16421","K16422","K16423","K16424","K16425","K16426","K16427","K16431",

"K16435","K16436","K16437","K16438","K16439","K16619","K16792","K16793","K16817","K16818","K16860",

"K16881","K17054","K17055","K17056","K17058","K17059","K17069","K17078","K17103","K17104","K17105",

"K17194","K17211","K17212","K17217","K17360","K17450","K17475","K17476","K17497","K17625","K17626",

"K17643","K17644","K17645","K17646","K17647","K17648","K17649","K17650","K17651","K17652","K17717",

"K17744","K17746","K17747","K17753","K17819","K17825","K17826","K17827","K17829","K17830","K17832",

"K17835","K17836","K17841","K17842","K17872","K17876","K17911","K17912","K17913","K17940","K17942",

"K17947","K17961","K17982","K17989","K18000","K18001","K18002","K18003","K18009","K18010","K18054",

"K18056","K18057","K18062","K18091","K18108","K18113","K18118","K18121","K18124","K18125","K18221",

"K18240","K18279","K18280","K18281","K18284","K18285","K18286","K18287","K18315","K18316","K18317",

"K18318","K18319","K18368","K18383","K18385","K18386","K18387","K18388","K18389","K18390","K18391",

"K18392","K18393","K18394","K18395","K18396","K18397","K18447","K18472","K18532","K18533","K18534",

"K18562","K18563","K18564","K18565","K18566","K18568","K18569","K18570","K18571","K18572","K18582",

"K18583","K18606","K18649","K18652","K18653","K18654","K18686","K18689","K18690","K18693","K18800",

"K18836","K18857","K18858","K18859","K18860","K18884","K18933","K18966","K19007","K19064","K19073",

"K19102","K19103","K19104","K19105","K19106","K19107","K19108","K19109","K19110","K19111","K19112",

"K19113","K19177","K19180","K19182","K19183","K19184","K19200","K19222","K19243","K19267","K19269",

"K19312","K19517","K19532","K19546","K19547","K19548","K19549","K19550","K19566","K19567","K19568",

"K19569","K19570","K19571","K19650","K19652","K19653","K19664","K19665","K19698","K19723","K19724",

"K19725","K19726","K19727","K19741","K19813","K19834","K19835","K19836","K19853","K19854","K19855",

"K19856","K19857","K19858","K19859","K19884","K19885","K19886","K19887","K19888","K19889","K19969",

"K19970","K19974","K19978","K19979","K19981","K19982","K20039","K20075","K20076","K20077","K20078",

"K20079","K20080","K20081","K20082","K20085","K20086","K20087","K20088","K20089","K20090","K20142",

"K20144","K20152","K20156","K20159","K20204","K20257","K20260","K20261","K20262","K20420","K20421",

"K20422","K20423","K20424","K20425","K20426","K20427","K20428","K20430","K20431","K20432","K20433","K20434",

"K20435","K20436","K20437","K20438","K20439","K20440","K20441","K20442","K20443","K20501","K20502",

"K20503","K20508","K20512","K20513","K20514","K20515","K20516","K20517","K20518","K20519","K20545",

"K20546","K20561","K20565","K20566","K20567","K20568","K20569","K20570","K20571","K20572","K20573",

"K20574","K20575","K20576","K20577","K20578","K20579","K20580","K20581","K20582","K20583","K20584",

"K20585","K20586","K20587","K20588","K20589","K20590","K20591","K20592","K20593","K20594","K20595",

"K20596","K20597","K20598","K20611","K20616","K20618","K20621","K20623","K20657","K20658","K20659",

"K20666","K20678","K20679","K20680","K20681","K20682","K20772","K20802","K20810","K20812","K20860",

"K20861","K20862","K20884","K20930","K20940","K20979","K20980","K20981","K20982","K20983","K20984",

"K20985","K20986","K21026","K21036","K21058","K21063","K21064","K21069","K21070","K21071","K21103",

"K21120","K21146","K21160","K21161","K21162","K21163","K21164","K21165","K21166","K21167","K21168",

"K21169","K21170","K21171","K21172","K21173","K21174","K21175","K21176","K21177","K21178","K21179",

"K21181","K21182","K21184","K21185","K21188","K21191","K21192","K21202","K21203","K21204","K21205",

"K21207","K21208","K21210","K21211","K21212","K21213","K21214","K21215","K21222","K21223","K21224",

"K21225","K21227","K21228","K21254","K21255","K21256","K21257","K21258","K21259","K21260","K21261",

"K21262","K21263","K21268","K21271","K21272","K21273","K21274","K21275","K21291","K21292","K21294",

"K21297","K21301","K21311","K21312","K21325","K21326","K21327","K21328","K21329","K21330","K21331",

"K21332","K21333","K21335","K21336","K21337","K21338","K21346","K21354","K21359","K21360","K21371",

"K21372","K21373","K21374","K21383","K21418","K21428","K21477","K21480","K21513","K21539","K21540",

"K21550","K21568","K21580","K21588","K21610","K21611","K21612","K21692","K21693","K21714","K21715",

"K21718","K21719","K21721","K21722","K21723","K21724","K21778","K21779","K21780","K21781","K21782",

"K21783","K21784","K21785","K21786","K21787","K21788","K21789","K21790","K21791","K21792","K21793",

"K21796","K21819","K21895","K21896","K21898","K21925","K21926","K21927","K21928","K21937","K21938",

"K21949","K22011","K22012","K22013","K22049","K22050","K22064","K22065","K22088","K22089","K22090",

"K22091","K22092","K22093","K22094","K22095","K22096","K22097","K22098","K22113","K22114","K22223",

"K22225","K22226","K22227","K22269","K22305","K22321","K22323","K22326","K22327","K22337","K22365",

"K22374","K22389","K22392","K22395","K22433","K22434","K22435","K22436","K22440","K22445","K22450",

"K22451","K22458","K22473","K22474","K22476","K22477","K22478","K22492","K22502","K22554","K22568",

"K22588","K22598","K22634","K22635","K22636","K22637","K22638","K22639","K22706","K22772","K22794",

"K22798","K22799","K22800","K22801","K22802","K22813","K22831","K22842","K22845","K22912","K22932",

"K22934","K22945","K22946","K22947","K22948","K22949","K22982","K22997","K22998","K23037","K23053",

"K23094","K23095","K23109","K23136","K23137","K23144","K23145","K23157","K23158","K23179","K23180",

"K23232","K23260","K23264","K23265","K23269","K23270","K23276","K23304","K23371","K23372","K23373",

"K23374","K23375","K23378","K23446","K23447","K23452","K23485","K23493","K23494","K23520","K23521",

"K23522","K23523","K23524","K23558","K23646","K23647","K23662","K23665","K23666","K23667","K23668",

"K23669","K23670","K23671","K23672","K23673","K23810","K23825","K23862","K23888","K23889","K23890",

"K23891","K23892","K23987","K23989","K23999","K24000","K24017","K24018","K24041","K24042","K24263",

"K24264","K24276","K24277","K24287","K24292","K24390","K24438","K24439","K24440","K24441","K24460",

"K24528","K24529","K24530","K24531","K24541","K24855","K24856","K24857","K24858","K24866","K24867",

"K24872","K24873","K25026","K25031","K25033","K25060",

"K25072","K25073","K25074","K25075","K25463","K25486","K25487","K25488","K25491","K25517","K25518",

"K25522","K25523","K25528","K25562","K25563","K25580","K25581","K25582","K25583","K25584","K25594",

"K25597","K25598","K25801","K25959","K25998","K26037","K26038")))

BSM<- cbind(rownames(BSM), data.frame(BSM, row.names=NULL))

names(BSM)[names(BSM) == 'rownames(BSM)'] <- 'Group'

BSMdata<-merge(EPFUmeta, BSM, by="Group")

BSMfuns<-BSMdata[15:955]

avgfBSMseason<-as.factor(BSMdata$season)

BSMfuns[BSMfuns > 0]<-1

betapart.core_BSMfun<-betapart.core(BSMfuns)

pair_funBSM<-beta.pair(betapart.core_BSMfun, index.family="sorensen")

SOR_funBSM<-(pair_funBSM$beta.sor)

SIM_funBSM<-(pair_funBSM$beta.sim)

SNE_funBSM<-(pair_funBSM$beta.sne)

betadisper_SOR_funBSM <-betadisper(SOR_funBSM, avgfBSMseason, type= c("centroid"), bias.adjust=FALSE, add=FALSE)

plot(betadisper_SOR_funBSM, axes=c(1,2), cex= 0.7, col = NULL, hull = TRUE, ellipse = FALSE, segments = TRUE, seg.col = "grey", label = FALSE, label.cex = 1, main= "")

(permutest.betadisper<-permutest(betadisper_SOR_funBSM, pairwise=FALSE, permutations=999, parallel=getOption("mc.cores"),))

boxplot(betadisper_SOR_funBSM, main="")

#non Sig

betadisper_SIM_funBSM <-betadisper(SIM_funBSM, avgfBSMseason, type= c("centroid"), bias.adjust=FALSE, add=FALSE)

plot(betadisper_SIM_funBSM, axes=c(1,2), cex= 0.7, col = NULL, hull = TRUE, ellipse = FALSE, segments = TRUE, seg.col = "grey", label = FALSE, label.cex = 1, main= "")

(permutest.betadisper<-permutest(betadisper_SIM_funBSM, pairwise=FALSE, permutations=999, parallel=getOption("mc.cores"),))

boxplot(betadisper_SIM_funBSM, main="")

#non Sig

betadisper_SNE_funBSM <-betadisper(SNE_funBSM, avgfBSMseason, type= c("centroid"), bias.adjust=FALSE, add=FALSE)

plot(betadisper_SNE_funBSM, axes=c(1,2), cex= 0.7, col = NULL, hull = TRUE, ellipse = FALSE, segments = TRUE, seg.col = "grey", label = FALSE, label.cex = 1, main= "")

(permutest.betadisper<-permutest(betadisper_SNE_funBSM, pairwise=FALSE, permutations=999, parallel=getOption("mc.cores"),))

boxplot(betadisper_SNE_funBSM, main="")

#non Sig

adonis2(SOR_funBSM~avgfBSMseason, data=BSMdata, permutations=999, method='bray')

#non

adonis2(SIM_funBSM~avgfBSMseason, data=BSMdata, permutations=999, method='bray')

#non

adonis2(SNE_funBSM~avgfBSMseason, data=BSMdata, permutations=999, method='bray')

#non

BSMAbund<-rowSums(BSMfuns)

BSMProp<-(BSMAbund/(ncol(EPFUfunfunctionRemove)))

shapiro.test(BSMProp[avgfBSMseason=="winter"])

shapiro.test(BSMProp[avgfBSMseason=="summer"])

plotNormalHistogram(BSMProp[avgfBSMseason=="winter"])

plotNormalHistogram(BSMProp[avgfBSMseason=="summer"])

bartlett.test(BSMProp~avgfBSMseason)

t.test(BSMProp ~ avgfBSMseason, data = BSMdata)

boxplot(BSMProp~avgfBSMseason)

p<-ggplot(BSMdata, aes(x=avgfBSMseason, y=BSMProp))+

stat_boxplot(geom='errorbar', linetype=1, width=0.5)+

geom_boxplot()+

xlab("")+

ylab("Gene Abundance")+

theme_classic()+

theme(axis.text=element_text(size=30))+

theme(text=element_text(size=40))

p

ggsave("WriteUp/Figs/240828_update/BSMabund.svg", width=15, height=10)

rm(betadisper_SIM_funBSM, betadisper_SNE_funBSM, betadisper_SOR_funMTP,

betapart.core_BSMfun, p, pair_funBSM, permutest.betadisper, res_aov, BSMAbund, BSMProp,

SIM_funBSM, SNE_funBSM, SOR_funBSM)

#####

#BSM across winters

#####

winterBSM<-subset(BSMdata, season=="winter")

winterBSMfuns<-winterBSM[15:955]

avgfBSMseason<-as.factor(winterBSM$yearseason )

winterBSMfuns[winterBSMfuns > 0]<-1

betapart.core_BSMfun_winter<-betapart.core(winterBSMfuns)

pair_funBSM_w<-beta.pair(betapart.core_BSMfun_winter, index.family="sorensen")

SOR_funBSM_winter<-(pair_funBSM_w$beta.sor)

SIM_funBSM_winter<-(pair_funBSM_w$beta.sim)

SNE_funBSM_winter<-(pair_funBSM_w$beta.sne)

betadisper_SOR_funMTP_w <-betadisper(SOR_funBSM_winter, avgfBSMseason, type= c("centroid"), bias.adjust=FALSE, add=FALSE)

plot(betadisper_SOR_funMTP_w, axes=c(1,2), cex= 0.7, col = NULL, hull = TRUE, ellipse = FALSE, segments = TRUE, seg.col = "grey", label = FALSE, label.cex = 1, main= "")

(permutest.betadisper<-permutest(betadisper_SOR_funMTP_w, pairwise=FALSE, permutations=999, parallel=getOption("mc.cores"),))

boxplot(betadisper_SOR_funMTP_w, main="")

#non Sig

betadisper_SIM_funMTP_w <-betadisper(SIM_funBSM_winter, avgfBSMseason, type= c("centroid"), bias.adjust=FALSE, add=FALSE)

plot(betadisper_SIM_funMTP_w, axes=c(1,2), cex= 0.7, col = NULL, hull = TRUE, ellipse = FALSE, segments = TRUE, seg.col = "grey", label = FALSE, label.cex = 1, main= "")

(permutest.betadisper<-permutest(betadisper_SIM_funMTP_w, pairwise=FALSE, permutations=999, parallel=getOption("mc.cores"),))

boxplot(betadisper_SIM_funMTP_w, main="")

#non Sig

betadisper_SNE_funMTP_w<-betadisper(SNE_funBSM_winter, avgfBSMseason, type= c("centroid"), bias.adjust=FALSE, add=FALSE)

plot(betadisper_SNE_funMTP_w, axes=c(1,2), cex= 0.7, col = NULL, hull = TRUE, ellipse = FALSE, segments = TRUE, seg.col = "grey", label = FALSE, label.cex = 1, main= "")

(permutest.betadisper<-permutest(betadisper_SNE_funMTP_w, pairwise=FALSE, permutations=999, parallel=getOption("mc.cores"),))

boxplot(betadisper_SNE_funMTP_w, main="")

#non Sig

adonis2(SOR_funBSM_winter~avgfBSMseason, data=winterBSM, permutations=999, method='bray')

#non

adonis2(SIM_funBSM_winter~avgfBSMseason, data=winterBSM, permutations=999, method='bray')

#non

adonis2(SNE_funBSM_winter~avgfBSMseason, data=winterBSM, permutations=999, method='bray')

#non

winterBSMfuns[winterBSMfuns > 0] <- 1

BSMWinterransAbund<-rowSums(winterBSMfuns)

BSMWinterProp<-(BSMWinterransAbund/(ncol(EPFUfunfunctionRemove)))

BSMwinterAbund<-rowSums(winterBSMfuns)

shapiro.test(BSMWinterProp[avgfBSMseason=="winter1"])

shapiro.test(BSMWinterProp[avgfBSMseason=="winter2"])

plotNormalHistogram(BSMWinterProp[avgfBSMseason=="winter1"])

plotNormalHistogram(BSMWinterProp[avgfBSMseason=="winter2"])

bartlett.test(BSMWinterProp~avgfBSMseason)

t.test(BSMWinterProp ~ avgfBSMseason, data = BSMdata)

boxplot(BSMWinterProp~avgfBSMseason)

p<-ggplot(winterBSM, aes(x=avgfBSMseason, y=BSMWinterProp))+

stat_boxplot(geom='errorbar', linetype=1, width=0.5)+

geom_boxplot()+

xlab("")+

ylab("Gene Abundance")+

theme_classic()+

theme(axis.text=element_text(size=30))+

theme(text=element_text(size=40))

p

rm(avgfBSMseason, BSM, BSMdata, BSMfuns, BSMwinterAbund, BSMWinterProp, BSMWinterransAbund,

m5, m6, MTP, MTransAbund, MTransProp, op, winterBSM, winterBSMfuns)

######

#Metabolic pathways

######

########

#Glycolysis

########

Gly<-EPFUfunfunctionRemove %>% select(any_of(c("K00001","K00002","K00016","K00114","K00121","K00128","K00129","K00131","K00134","K00138","K00149","K00150","K00161","K00162","K00163","K00169","K00170","K00171","K00172","K00174","K00175","K00189","K00382","K00627","K00844","K00845","K00850","K00873","K00886","K00895","K00918","K00927","K01006","K01007","K01084","K01085","K01086","K01222","K01223","K01568","K01596","K01610","K01622","K01623","K01624","K01689","K01785","K01792","K01803","K01810","K01834","K01835","K01837","K01895","K01905","K01913","K02446","K02753","K02777","K02779","K02791","K03103","K03737","K03841","K04022","K04041","K04072","K06859","K08074","K10705","K11389","K11532","K11645","K12406","K12407","K12957","K13810","K13951","K13952","K13953","K13954","K13979","K13980","K14028","K14029","K14085","K15633","K15634","K15635","K15778","K15779","K15916","K16305","K16306","K16370","K18857","K18978","K20118","K20866","K21071","K22224","K22473","K22474","K24012","K24182","K25026")))

Gly<- cbind(rownames(Gly), data.frame(Gly, row.names=NULL))

names(Gly)[names(Gly) == 'rownames(Gly)'] <- 'Group'

Glydata<-merge(EPFUmeta, Gly, by="Group")

Glyfuns<-Glydata[15:98]

Glyseason<-as.factor(Glydata$season)

Glyfuns[Glyfuns > 0]<-1

betapart.core_Pglyfuns<-betapart.core(Glyfuns)

pair_funGlyfuns<-beta.pair(betapart.core_Pglyfuns, index.family="sorensen")

SOR_funGly<-(pair_funGlyfuns$beta.sor)

SIM_funGly<-(pair_funGlyfuns$beta.sim)

SNE_funGly<-(pair_funGlyfuns$beta.sne)

betadisper_SOR_fungly <-betadisper(SOR_funGly, Glyseason, type= c("centroid"), bias.adjust=FALSE, add=FALSE)

plot(betadisper_SOR_fungly, axes=c(1,2), cex= 0.7, col = NULL, hull = TRUE, ellipse = FALSE, segments = TRUE, seg.col = "grey", label = FALSE, label.cex = 1, main= "")

(permutest.betadisper<-permutest(betadisper_SOR_fungly, pairwise=FALSE, permutations=999, parallel=getOption("mc.cores"),))

boxplot(betadisper_SOR_fungly, main="")

#non Sig

betadisper_SIM_fungly <-betadisper(SIM_funGly, Glyseason, type= c("centroid"), bias.adjust=FALSE, add=FALSE)

plot(betadisper_SIM_fungly, axes=c(1,2), cex= 0.7, col = NULL, hull = TRUE, ellipse = FALSE, segments = TRUE, seg.col = "grey", label = FALSE, label.cex = 1, main= "")

(permutest.betadisper<-permutest(betadisper_SIM_fungly, pairwise=FALSE, permutations=999, parallel=getOption("mc.cores"),))

boxplot(betadisper_SIM_fungly, main="")

#non Sig

betadisper_SNE_fungly <-betadisper(SNE_funGly, Glyseason, type= c("centroid"), bias.adjust=FALSE, add=FALSE)

plot(betadisper_SNE_fungly, axes=c(1,2), cex= 0.7, col = NULL, hull = TRUE, ellipse = FALSE, segments = TRUE, seg.col = "grey", label = FALSE, label.cex = 1, main= "")

(permutest.betadisper<-permutest(betadisper_SNE_fungly, pairwise=FALSE, permutations=999, parallel=getOption("mc.cores"),))

boxplot(betadisper_SNE_fungly, main="")

#non Sig

adonis2(SOR_funGly~Glyseason, data=Glydata, permutations=999, method='bray')

#non

adonis2(SIM_funGly~Glyseason, data=Glydata, permutations=999, method='bray')

#non

adonis2(SNE_funGly~Glyseason, data=Glydata, permutations=999, method='bray')

#non

GlyAbund<-rowSums(Glyfuns)

GlyProp<-(GlyAbund/(ncol(EPFUfunfunctionRemove)))

shapiro.test(GlyProp[Glyseason=="winter"])

shapiro.test(GlyProp[Glyseason=="summer"])

plotNormalHistogram(GlyProp[Glyseason=="winter"])

plotNormalHistogram(GlyProp[Glyseason=="summer"])

kruskal.test(GlyProp ~ Glyseason, data = Glydata)

boxplot(GlyAbund~Glyseason)

p<-ggplot(Glydata, aes(x=Glyseason, y=GlyProp))+

stat_boxplot(geom='errorbar', linetype=1, width=0.5)+

geom_boxplot()+

geom_text(x=1, y=155, label="a", size=20)+

geom_text(x=2, y=155, label="b", size=20)+

xlab("")+

ylab("Gene Abundance")+

#ylim(80,160)+

theme_classic()+

theme(axis.text=element_text(size=30))+

theme(text=element_text(size=40))

p

ggsave("WriteUp/Figs/240828_update/Glycolysis.svg", width=15, height=10)

rm(betadisper_SIM_fungly, betadisper_SNE_fungly, betadisper_SOR_fungly,

betapart.core_Pglyfuns, Glyfuns, Gly, m6, op, p, pair_funGlyfuns,

permutest.betadisper, GlyAbund, GlyProp, Glyseason, SIM_funGly,SNE_funGly, SOR_funGly)

######

#Gly winters

#####

wintergly<-subset(Glydata, season=="winter")

winterGlyfuns<-wintergly[15:98]

Glyyear<-as.factor(wintergly$yearseason)

winterGlyfuns[winterGlyfuns > 0]<-1

betapart.core_Pglyfuns_winter<-betapart.core(winterGlyfuns)

pair_funGlyfuns_winter<-beta.pair(betapart.core_Pglyfuns_winter, index.family="sorensen")

SOR_funGly_winter<-(pair_funGlyfuns_winter$beta.sor)

SIM_funGly_winter<-(pair_funGlyfuns_winter$beta.sim)

SNE_funGly_winter<-(pair_funGlyfuns_winter$beta.sne)

betadisper_SOR_fungly_winter <-betadisper(SOR_funGly_winter, Glyyear, type= c("centroid"), bias.adjust=FALSE, add=FALSE)

plot(betadisper_SOR_fungly_winter, axes=c(1,2), cex= 0.7, col = NULL, hull = TRUE, ellipse = FALSE, segments = TRUE, seg.col = "grey", label = FALSE, label.cex = 1, main= "")

(permutest.betadisper<-permutest(betadisper_SOR_fungly_winter, pairwise=FALSE, permutations=999, parallel=getOption("mc.cores"),))

boxplot(betadisper_SOR_fungly_winter, main="")

#non Sig

betadisper_SIM_fungly_winter <-betadisper(SIM_funGly_winter, Glyyear, type= c("centroid"), bias.adjust=FALSE, add=FALSE)

plot(betadisper_SIM_fungly_winter, axes=c(1,2), cex= 0.7, col = NULL, hull = TRUE, ellipse = FALSE, segments = TRUE, seg.col = "grey", label = FALSE, label.cex = 1, main= "")

(permutest.betadisper<-permutest(betadisper_SIM_fungly_winter, pairwise=FALSE, permutations=999, parallel=getOption("mc.cores"),))

boxplot(betadisper_SIM_fungly_winter, main="")

#non Sig

betadisper_SNE_fungly_winter <-betadisper(SIM_funGly_winter, Glyyear, type= c("centroid"), bias.adjust=FALSE, add=FALSE)

plot(betadisper_SNE_fungly_winter, axes=c(1,2), cex= 0.7, col = NULL, hull = TRUE, ellipse = FALSE, segments = TRUE, seg.col = "grey", label = FALSE, label.cex = 1, main= "")

(permutest.betadisper<-permutest(betadisper_SNE_fungly_winter, pairwise=FALSE, permutations=999, parallel=getOption("mc.cores"),))

boxplot(betadisper_SNE_fungly_winter, main="")

#non Sig

adonis2(SOR_funGly_winter~Glyyear, data=Glydata, permutations=999, method='bray')

#non

adonis2(SIM_funGly_winter~Glyyear, data=Glydata, permutations=999, method='bray')

#non

adonis2(SNE_funGly_winter~Glyyear, data=Glydata, permutations=999, method='bray')

#non

winterGlyAbund<-rowSums(winterGlyfuns)

winterGlyProp<-(winterGlyAbund/(ncol(EPFUfunfunctionRemove)))

shapiro.test(winterGlyProp[Glyyear=="winter1"])

shapiro.test(winterGlyProp[Glyyear=="winter2"])

plotNormalHistogram(winterGlyProp[Glyyear=="winter1"])

plotNormalHistogram(winterGlyProp[Glyyear=="winter2"])

bartlett.test(winterGlyProp~Glyyear)

t.test(winterGlyProp ~ Glyyear, data = Glydata)

boxplot(winterGlyAbund~Glyyear)

p<-ggplot(Glydata, aes(x=winterGlyAbund, y=Glyyear))+

stat_boxplot(geom='errorbar', linetype=1, width=0.5)+

geom_boxplot()+

geom_text(x=1, y=155, label="a", size=20)+

geom_text(x=2, y=155, label="b", size=20)+

xlab("")+

ylab("Gene Abundance")+

#ylim(80,160)+

theme_classic()+

theme(axis.text=element_text(size=30))+

theme(text=element_text(size=40))

p

ggsave(".svg", width=15, height=10)

rm(betadisper_SIM_fungly_winter, betadisper_SNE_fungly_winter, betadisper_SOR_fungly_winter,

betapart.core_Pglyfuns_winter, Glydata, m6, op, p ,pair_funGlyfuns_winter, permutest.betadisper,

wintergly, winterGlyfuns, Glyyear, SIM_funGly_winter, SOR_funGly_winter,

winterGlyAbund, winterGlyProp, SNE_funGly_winter)

########

#Oxidative Phosphor

########

Oxidative<-EPFUfunfunctionRemove %>% select(any_of(c("K00233","K00234","K00235","K00236","K00237","K00239","K00240","K00241","K00242","K00244","K00245","K00246","K00247","K00330","K00331","K00332","K00333","K00334","K00335","K00336","K00337","K00338","K00339","K00340","K00341","K00342","K00343","K00404","K00405","K00406","K00407","K00410","K00411","K00412","K00413","K00414","K00415","K00416","K00417","K00418","K00419","K00420","K00424","K00425","K00426","K00937","K01507","K01535","K01542","K01543","K01544","K01549","K02107","K02108","K02109","K02110","K02111","K02112","K02113","K02114","K02115","K02117","K02118","K02119","K02120","K02121","K02122","K02123","K02124","K02125","K02126","K02127","K02128","K02129","K02130","K02131","K02132","K02133","K02134","K02135","K02136","K02137","K02138","K02139","K02140","K02141","K02142","K02143","K02144","K02145","K02146","K02147","K02148","K02149","K02150","K02151","K02152","K02153","K02154","K02155","K02256","K02257","K02258","K02259","K02260","K02261","K02262","K02263","K02264","K02265","K02266","K02267","K02268","K02269","K02270","K02271","K02272","K02273","K02274","K02275","K02276","K02277","K02297","K02298","K02299","K02300","K02826","K02827","K02828","K02829","K03661","K03662","K03878","K03879","K03880","K03881","K03882","K03883","K03884","K03885","K03886","K03887","K03888","K03889","K03890","K03891","K03934","K03935","K03936","K03937","K03938","K03939","K03940","K03941","K03942","K03943","K03944","K03945","K03946","K03947","K03948","K03949","K03950","K03951","K03952","K03953","K03954","K03955","K03956","K03957","K03958","K03959","K03960","K03961","K03962","K03963","K03964","K03965","K03966","K03967","K03968","K05572","K05573","K05574","K05575","K05576","K05577","K05578","K05579","K05580","K05581","K05582","K05583","K05584","K05585","K05586","K05587","K05588","K06019","K08738","K11351","K11352","K11353","K11725","K11726","K13378","K13380","K15408","K15862","K15863","K15986","K18859","K18860","K22468","K22501","K24007","K24008","K24009","K24010","K24011","K25801","K25995","K25996")))

Oxidative<- cbind(rownames(Oxidative), data.frame(Oxidative, row.names=NULL))

names(Oxidative)[names(Oxidative) == 'rownames(Oxidative)'] <- 'Group'

Oxidata<-merge(EPFUmeta, Oxidative, by="Group")

Oxifuns<-Oxidata[15:114]

Oxiseason<-as.factor(Oxidata$season)

Oxifuns[Oxifuns > 0]<-1

betapart.core_Oxifuns<-betapart.core(Oxifuns)

pair_funOxifuns<-beta.pair(betapart.core_Oxifuns, index.family="sorensen")

SOR_funOxi<-(pair_funOxifuns$beta.sor)

SIM_funOxi<-(pair_funOxifuns$beta.sim)

SNE_funOxi<-(pair_funOxifuns$beta.sne)

betadisper_SOR_funOxi <-betadisper(SOR_funOxi, Oxiseason, type= c("centroid"), bias.adjust=FALSE, add=FALSE)

plot(betadisper_SOR_funOxi, axes=c(1,2), cex= 0.7, col = NULL, hull = TRUE, ellipse = FALSE, segments = TRUE, seg.col = "grey", label = FALSE, label.cex = 1, main= "")

(permutest.betadisper<-permutest(betadisper_SOR_funOxi, pairwise=FALSE, permutations=999, parallel=getOption("mc.cores"),))

boxplot(betadisper_SOR_funOxi, main="")

#non Sig

betadisper_SIM_funOxi <-betadisper(SIM_funOxi, Oxiseason, type= c("centroid"), bias.adjust=FALSE, add=FALSE)

plot(betadisper_SIM_funOxi, axes=c(1,2), cex= 0.7, col = NULL, hull = TRUE, ellipse = FALSE, segments = TRUE, seg.col = "grey", label = FALSE, label.cex = 1, main= "")

(permutest.betadisper<-permutest(betadisper_SIM_funOxi, pairwise=FALSE, permutations=999, parallel=getOption("mc.cores"),))

boxplot(betadisper_SIM_funOxi, main="")

#non Sig

betadisper_SNE_funOxi <-betadisper(SNE_funOxi, Oxiseason, type= c("centroid"), bias.adjust=FALSE, add=FALSE)

plot(betadisper_SNE_funOxi, axes=c(1,2), cex= 0.7, col = NULL, hull = TRUE, ellipse = FALSE, segments = TRUE, seg.col = "grey", label = FALSE, label.cex = 1, main= "")

(permutest.betadisper<-permutest(betadisper_SNE_funOxi, pairwise=FALSE, permutations=999, parallel=getOption("mc.cores"),))

boxplot(betadisper_SNE_funOxi, main="")

#non Sig

adonis2(SOR_funOxi~Oxiseason, data=Oxidata, permutations=999, method='bray')

#non

adonis2(SIM_funOxi~Oxiseason, data=Oxidata, permutations=999, method='bray')

#non

adonis2(SNE_funOxi~Oxiseason, data=Oxidata, permutations=999, method='bray')

#non

OxiAbund<-rowSums(Oxifuns)

OxiProp<-(OxiAbund/(ncol(EPFUfunfunctionRemove)))

shapiro.test(OxiProp[Oxiseason=="winter"])

shapiro.test(OxiProp[Oxiseason=="summer"])

plotNormalHistogram(OxiProp[Oxiseason=="winter"])

plotNormalHistogram(OxiProp[Oxiseason=="summer"])

kruskal.test(OxiProp ~ Oxiseason, data = Oxidata)

boxplot(OxiProp~Oxiseason)

p<-ggplot(Oxidata, aes(x=Oxiseason, y=OxiProp))+

stat_boxplot(geom='errorbar', linetype=1, width=0.5)+

geom_boxplot()+

# geom_text(x=1, y=155, label="a", size=20)+

#geom_text(x=2, y=155, label="b", size=20)+

xlab("")+

ylab("Gene Abundance")+

#ylim(80,160)+

theme_classic()+

theme(axis.text=element_text(size=30))+

theme(text=element_text(size=40))

p

ggsave("WriteUp/Figs/240828_update/OxiPhos.svg", width=15, height=10)

rm(betadisper_SIM_funOxi, betadisper_SNE_funOxi, betadisper_SOR_funOxi,

betapart.core_Oxifuns, m6, op, Oxidative, pair_funOxifuns, permutest.betadisper,

OxiAbund,OxiProp, Oxiseason, SIM_funOxi, SNE_funOxi, SOR_funOxi, Oxifuns )

######

#Oxidataive Phos Winters

#####

oxiwinter<-subset(Oxidata, season=="winter")

oxiwinterfuns<-oxiwinter[15:114]

Oxiyear<-as.factor(oxiwinter$yearseason)

oxiwinterfuns[oxiwinterfuns > 0]<-1

betapart.core_Oxi_winter<-betapart.core(oxiwinterfuns)

pair_funOxi_winter<-beta.pair(betapart.core_Oxi_winter, index.family="sorensen")

SOR_funOxi_winter<-(pair_funOxi_winter$beta.sor)

SIM_funOxi_winter<-(pair_funOxi_winter$beta.sim)

SNE_funOxi_winter<-(pair_funOxi_winter$beta.sne)

betadisper_SOR_Oxi_winter <-betadisper(SOR_funOxi_winter, Oxiyear, type= c("centroid"), bias.adjust=FALSE, add=FALSE)

plot(betadisper_SOR_Oxi_winter, axes=c(1,2), cex= 0.7, col = NULL, hull = TRUE, ellipse = FALSE, segments = TRUE, seg.col = "grey", label = FALSE, label.cex = 1, main= "")

(permutest.betadisper<-permutest(betadisper_SOR_Oxi_winter, pairwise=FALSE, permutations=999, parallel=getOption("mc.cores"),))

boxplot(betadisper_SOR_Oxi_winter, main="")

#non Sig

betadisper_SIM_Oxi_winter <-betadisper(SIM_funOxi_winter, Oxiyear, type= c("centroid"), bias.adjust=FALSE, add=FALSE)

plot(betadisper_SIM_Oxi_winter, axes=c(1,2), cex= 0.7, col = NULL, hull = TRUE, ellipse = FALSE, segments = TRUE, seg.col = "grey", label = FALSE, label.cex = 1, main= "")

(permutest.betadisper<-permutest(betadisper_SIM_Oxi_winter, pairwise=FALSE, permutations=999, parallel=getOption("mc.cores"),))

boxplot(betadisper_SIM_Oxi_winter, main="")

#non Sig

betadisper_SNE_Oxi_winter <-betadisper(SNE_funOxi_winter, Oxiyear, type= c("centroid"), bias.adjust=FALSE, add=FALSE)

plot(betadisper_SNE_Oxi_winter, axes=c(1,2), cex= 0.7, col = NULL, hull = TRUE, ellipse = FALSE, segments = TRUE, seg.col = "grey", label = FALSE, label.cex = 1, main= "")

(permutest.betadisper<-permutest(betadisper_SNE_Oxi_winter, pairwise=FALSE, permutations=999, parallel=getOption("mc.cores"),))

boxplot(betadisper_SNE_Oxi_winter, main="")

#non Sig

adonis2(SOR_funOxi_winter~Oxiyear, data=oxiwinter, permutations=999, method='bray')

#non

adonis2(SIM_funOxi_winter~Oxiyear, data=oxiwinter, permutations=999, method='bray')

#non

adonis2(SNE_funOxi_winter~Oxiyear, data=oxiwinter, permutations=999, method='bray')

#non

OxiWinterAbund<-rowSums(oxiwinterfuns)

OxiwinterProp<-(OxiWinterAbund/(ncol(EPFUfunfunctionRemove)))

shapiro.test(OxiwinterProp[Oxiyear=="winter1"])

shapiro.test(OxiwinterProp[Oxiyear=="winter2"])

plotNormalHistogram(OxiwinterProp[Oxiyear=="winter1"])

plotNormalHistogram(OxiwinterProp[Oxiyear=="winter2"])

bartlett.test(OxiwinterProp~Oxiyear)

t.test(OxiwinterProp ~ Oxiyear, data = oxiwinter)

boxplot(OxiwinterProp~Oxiyear)

p<-ggplot(oxiwinter, aes(x=Oxiyear, y=OxiWinterAbund))+

stat_boxplot(geom='errorbar', linetype=1, width=0.5)+

geom_boxplot()+

geom_text(x=1, y=155, label="a", size=20)+

geom_text(x=2, y=155, label="b", size=20)+

xlab("")+

ylab("Gene Abundance")+

#ylim(80,160)+

theme_classic()+

theme(axis.text=element_text(size=30))+

theme(text=element_text(size=40))

p

ggsave(".svg", width=15, height=10)

rm(betadisper_SIM_Oxi_winter, betadisper_SNE_Oxi_winter, betadisper_SOR_Oxi_winter,

betapart.core_Oxi_winter, m6, op, p ,Oxidata, oxiwinter, pair_funOxi_winter, permutest.betadisper,

OxiWinterAbund, OxiwinterPropm ,Oxiyear, oxiwinterfuns, SIM_funOxi_winter, SNE_funOxi_winter, SOR_funOxi_winter, OxiwinterProp)

########

#Methane

########

Methane<-EPFUfunfunctionRemove %>% select(any_of(c("K00018","K00024","K00058","K00093","K00121","K00122","K00123","K00124","K00125","K00126","K00127","K00148","K00169","K00170","K00171","K00172","K00189","K00192","K00193","K00194","K00195","K00196","K00197","K00198","K00200","K00201","K00202","K00203","K00204","K00205","K00300","K00317","K00319","K00320","K00399","K00400","K00401","K00402","K00440","K00441","K00442","K00443","K00577","K00578","K00579","K00580","K00581","K00582","K00583","K00584","K00600","K00625","K00672","K00830","K00831","K00850","K00863","K00918","K00925","K01007","K01070","K01079","K01086","K01499","K01595","K01622","K01623","K01624","K01689","K01834","K01895","K02203","K02446","K03388","K03389","K03390","K03396","K03421","K03422","K03532","K03533","K03841","K04041","K04480","K05299","K05884","K05979","K06034","K06914","K07072","K07144","K07811","K07812","K07821","K08093","K08094","K08097","K08264","K08265","K08685","K08691","K08692","K09733","K10713","K10714","K10944","K10945","K10946","K10977","K10978","K11212","K11260","K11261","K11529","K11532","K11645","K11779","K11780","K11781","K12234","K13039","K13788","K13812","K13831","K13942","K14028","K14029","K14067","K14080","K14081","K14082","K14083","K14084","K14126","K14127","K14128","K14940","K14941","K15022","K15228","K15229","K15633","K15634","K15635","K16157","K16158","K16159","K16160","K16161","K16162","K16176","K16177","K16178","K16179","K16254","K16255","K16256","K16257","K16258","K16259","K16260","K16305","K16306","K16370","K16792","K16793","K17066","K17067","K17068","K17100","K18277","K18933","K19793","K21071","K22015","K22081","K22082","K22083","K22084","K22085","K22086","K22087","K22305","K22480","K22481","K22482","K22515","K22516","K23995","K24182","K24393","K24998","K25123","K25124","K25528")))

Methane<- cbind(rownames(Methane), data.frame(Methane, row.names=NULL))

names(Methane)[names(Methane) == 'rownames(Methane)'] <- 'Group'

metdata<-merge(EPFUmeta, Methane, by="Group")

Metfuns<-metdata[15:154]

Metseason<-as.factor(metdata$season)

Metfuns[Metfuns > 0]<-1

betapart.core_Metfuns<-betapart.core(Metfuns)

pair_funMetfuns<-beta.pair(betapart.core_Metfuns, index.family="sorensen")

SOR_funMet<-(pair_funMetfuns$beta.sor)

SIM_funMet<-(pair_funMetfuns$beta.sim)

SNE_funMet<-(pair_funMetfuns$beta.sne)

betadisper_SOR_funMet <-betadisper(SOR_funMet, Metseason, type= c("centroid"), bias.adjust=FALSE, add=FALSE)

plot(betadisper_SOR_funMet, axes=c(1,2), cex= 0.7, col = NULL, hull = TRUE, ellipse = FALSE, segments = TRUE, seg.col = "grey", label = FALSE, label.cex = 1, main= "")

(permutest.betadisper<-permutest(betadisper_SOR_funMet, pairwise=FALSE, permutations=999, parallel=getOption("mc.cores"),))

boxplot(betadisper_SOR_funMet, main="")

#non Sig

betadisper_SIM_funMet <-betadisper(SIM_funMet, Metseason, type= c("centroid"), bias.adjust=FALSE, add=FALSE)

plot(betadisper_SIM_funMet, axes=c(1,2), cex= 0.7, col = NULL, hull = TRUE, ellipse = FALSE, segments = TRUE, seg.col = "grey", label = FALSE, label.cex = 1, main= "")

(permutest.betadisper<-permutest(betadisper_SIM_funMet, pairwise=FALSE, permutations=999, parallel=getOption("mc.cores"),))

boxplot(betadisper_SIM_funMet, main="")

#non Sig

betadisper_SNE_funMet <-betadisper(SNE_funMet, Metseason, type= c("centroid"), bias.adjust=FALSE, add=FALSE)

plot(betadisper_SNE_funMet, axes=c(1,2), cex= 0.7, col = NULL, hull = TRUE, ellipse = FALSE, segments = TRUE, seg.col = "grey", label = FALSE, label.cex = 1, main= "")

(permutest.betadisper<-permutest(betadisper_SNE_funMet, pairwise=FALSE, permutations=999, parallel=getOption("mc.cores"),))

boxplot(betadisper_SNE_funMet, main="")

#non Sig

adonis2(SOR_funMet~Metseason, data=metdata, permutations=999, method='bray')

#non

adonis2(SIM_funMet~Metseason, data=metdata, permutations=999, method='bray')

#non

adonis2(SNE_funMet~Metseason, data=metdata, permutations=999, method='bray')

#non

MetAbund<-rowSums(Metfuns)

MEtProp<-(MetAbund/(ncol(EPFUfunfunctionRemove)))

shapiro.test(MEtProp[Metseason=="winter"])

shapiro.test(MEtProp[Metseason=="summer"])

plotNormalHistogram(MEtProp[Metseason=="winter"])

plotNormalHistogram(MEtProp[Metseason=="summer"])

kruskal.test(MEtProp ~ Metseason, data = metdata)

boxplot(MetAbund~Metseason)

p<-ggplot(metdata, aes(x=Metseason, y=MEtProp))+

stat_boxplot(geom='errorbar', linetype=1, width=0.5)+

geom_boxplot()+

# geom_text(x=1, y=155, label="a", size=20)+

#geom_text(x=2, y=155, label="b", size=20)+

xlab("")+

ylab("Gene Abundance")+

#ylim(80,160)+

theme_classic()+

theme(axis.text=element_text(size=30))+

theme(text=element_text(size=40))

p

ggsave("WriteUp/Figs/240828_update/Methane.svg", width=15, height=10)

rm(betadisper_SIM_funMet,betadisper_SNE_funMet,betadisper_SOR_funMet,betapart.core_Metfuns,

m6,MetAbund,Metfuns,Methane,MEtProp,Metseason, op,pair_funMetfuns,

permutest.betadisper,SIM_funMet,SNE_funMet,SOR_funMet )

######

#Methane Winters

#####

Metwinter<-subset(metdata, season=="winter")

Metwinterfuns<-Metwinter[15:ncol(Metwinter)]

Metyear<-as.factor(Metwinter$yearseason)

Metwinterfuns[Metwinterfuns > 0]<-1

betapart.core_Metwinter<-betapart.core(Metwinterfuns)

pair_fun_Metwinter<-beta.pair(betapart.core_Metwinter, index.family="sorensen")

SOR_fun_Metwinter<-(pair_fun_Metwinter$beta.sor)

SIM_fun_Metwinter<-(pair_fun_Metwinter$beta.sim)

SNE_fun_Metwinter<-(pair_fun_Metwinter$beta.sne)

betadisper_SOR__Metwinter <-betadisper(SOR_fun_Metwinter, Metyear, type= c("centroid"), bias.adjust=FALSE, add=FALSE)

plot(betadisper_SOR__Metwinter, axes=c(1,2), cex= 0.7, col = NULL, hull = TRUE, ellipse = FALSE, segments = TRUE, seg.col = "grey", label = FALSE, label.cex = 1, main= "")

(permutest.betadisper<-permutest(betadisper_SOR__Metwinter, pairwise=FALSE, permutations=999, parallel=getOption("mc.cores"),))

boxplot(betadisper_SOR__Metwinter, main="")

#non Sig

betadisper_SIM__Metwinter <-betadisper(SIM_fun_Metwinter, Metyear, type= c("centroid"), bias.adjust=FALSE, add=FALSE)

plot(betadisper_SIM__Metwinter, axes=c(1,2), cex= 0.7, col = NULL, hull = TRUE, ellipse = FALSE, segments = TRUE, seg.col = "grey", label = FALSE, label.cex = 1, main= "")

(permutest.betadisper<-permutest(betadisper_SIM__Metwinter, pairwise=FALSE, permutations=999, parallel=getOption("mc.cores"),))

boxplot(betadisper_SIM__Metwinter, main="")

#non Sig

betadisper_SNE__Metwinter <-betadisper(SNE_fun_Metwinter, Metyear, type= c("centroid"), bias.adjust=FALSE, add=FALSE)

plot(betadisper_SNE__Metwinter, axes=c(1,2), cex= 0.7, col = NULL, hull = TRUE, ellipse = FALSE, segments = TRUE, seg.col = "grey", label = FALSE, label.cex = 1, main= "")

(permutest.betadisper<-permutest(betadisper_SNE__Metwinter, pairwise=FALSE, permutations=999, parallel=getOption("mc.cores"),))

boxplot(betadisper_SNE__Metwinter, main="")

#non Sig

adonis2(SOR_fun_Metwinter~Metyear, data=Metwinter, permutations=999, method='bray')

#non

adonis2(SIM_fun_Metwinter~Metyear, data=Metwinter, permutations=999, method='bray')

#non

adonis2(SNE_fun_Metwinter~Metyear, data=Metwinter, permutations=999, method='bray')

#non

MetWinterAbund<-rowSums(Metwinterfuns)

MetwinterProp<-(MetWinterAbund/(ncol(EPFUfunfunctionRemove)))

shapiro.test(MetwinterProp[Metyear=="winter1"])

shapiro.test(MetwinterProp[Metyear=="winter2"])

plotNormalHistogram(MetwinterProp[Metyear=="winter1"])

plotNormalHistogram(MetwinterProp[Metyear=="winter2"])

kruskal.test(MetwinterProp ~ Metyear, data = metdata)

boxplot(MetwinterProp~Metyear)

p<-ggplot(Metwinter, aes(x=Metyear, y=MetWinterAbund))+

stat_boxplot(geom='errorbar', linetype=1, width=0.5)+

geom_boxplot()+

geom_text(x=1, y=155, label="a", size=20)+

geom_text(x=2, y=155, label="b", size=20)+

xlab("")+

ylab("Gene Abundance")+

#ylim(80,160)+

theme_classic()+

theme(axis.text=element_text(size=30))+

theme(text=element_text(size=40))

p

ggsave(".svg", width=15, height=10)

rm(betadisper_SIM__Metwinter,betadisper_SNE__Metwinter,betadisper_SOR__Metwinter,

betapart.core_Metwinter,m6,metdata,Metwinter,MetWinterAbund,Metwinterfuns,

MetwinterProp,Metyear, op, p, pair_fun_Metwinter, permutest.betadisper, SIM_fun_Metwinter, SNE_fun_Metwinter,

SOR_fun_Metwinter)

########

#Sulfur

########

Sulfur<-EPFUfunfunctionRemove %>% select(any_of(c("K00184","K00185","K00299","K00380","K00381","K00385","K00386","K00387","K00390","K00392","K00394","K00395","K00640","K00641","K00651","K00860","K00955","K00956","K00957","K00958","K00988","K01011","K01082","K01738","K01739","K02045","K02046","K02047","K02048","K02439","K03119","K04091","K05301","K05907","K05908","K06881","K07306","K07307","K07308","K08352","K08353","K08354","K08357","K08358","K08359","K10150","K10764","K10831","K11180","K11181","K13034","K13811","K15422","K15551","K15552","K15553","K15554","K15555","K15759","K15762","K15765","K16936","K16937","K16950","K16951","K16952","K16953","K16954","K16955","K16964","K16965","K16966","K16967","K16968","K16969","K17069","K17217","K17218","K17219","K17220","K17221","K17222","K17223","K17224","K17225","K17226","K17227","K17228","K17229","K17230","K17285","K17486","K17725","K17993","K17994","K17995","K17996","K20034","K20035","K20036","K21307","K21308","K21309","K21310","K22470","K22622","K22966","K23163","K23304")))

Sulfur<- cbind(rownames(Sulfur), data.frame(Sulfur, row.names=NULL))

names(Sulfur)[names(Sulfur) == 'rownames(Sulfur)'] <- 'Group'

Suldata<-merge(EPFUmeta, Sulfur, by="Group")

Sulfuns<-Suldata[15:94]

Sulseason<-as.factor(Suldata$season)

Sulfuns[Sulfuns > 0]<-1

betapart.core_Sulfuns<-betapart.core(Sulfuns)

pair_funSulfuns<-beta.pair(betapart.core_Sulfuns, index.family="sorensen")

SOR_funSul<-(pair_funSulfuns$beta.sor)

SIM_funSul<-(pair_funSulfuns$beta.sim)

SNE_funSul<-(pair_funSulfuns$beta.sne)

betadisper_SOR_funSul <-betadisper(SOR_funSul, Sulseason, type= c("centroid"), bias.adjust=FALSE, add=FALSE)

plot(betadisper_SOR_funSul, axes=c(1,2), cex= 0.7, col = NULL, hull = TRUE, ellipse = FALSE, segments = TRUE, seg.col = "grey", label = FALSE, label.cex = 1, main= "")

(permutest.betadisper<-permutest(betadisper_SOR_funSul, pairwise=FALSE, permutations=999, parallel=getOption("mc.cores"),))

boxplot(betadisper_SOR_funSul, main="")

#non Sig

betadisper_SIM_funSul <-betadisper(SIM_funSul, Sulseason, type= c("centroid"), bias.adjust=FALSE, add=FALSE)

plot(betadisper_SIM_funNit, axes=c(1,2), cex= 0.7, col = NULL, hull = TRUE, ellipse = FALSE, segments = TRUE, seg.col = "grey", label = FALSE, label.cex = 1, main= "")

(permutest.betadisper<-permutest(betadisper_SIM_funSul, pairwise=FALSE, permutations=999, parallel=getOption("mc.cores"),))

boxplot(betadisper_SIM_funSul, main="")

#non Sig

betadisper_SNE_funSul <-betadisper(SNE_funSul,Sulseason, type= c("centroid"), bias.adjust=FALSE, add=FALSE)

plot(betadisper_SNE_funSul, axes=c(1,2), cex= 0.7, col = NULL, hull = TRUE, ellipse = FALSE, segments = TRUE, seg.col = "grey", label = FALSE, label.cex = 1, main= "")

(permutest.betadisper<-permutest(betadisper_SNE_funSul, pairwise=FALSE, permutations=999, parallel=getOption("mc.cores"),))

boxplot(betadisper_SNE_funSul, main="")

#non Sig

adonis2(SOR_funSul~Sulseason, data=Suldata, permutations=999, method='bray')

#non

adonis2(SIM_funSul~Sulseason, data=Suldata, permutations=999, method='bray')

#non

adonis2(SNE_funSul~Sulseason, data=Suldata, permutations=999, method='bray')

#non

SulAbund<-rowSums(Sulfuns)

SulProp<-(SulAbund/(ncol(EPFUfunfunctionRemove)))

shapiro.test(SulProp[Sulseason=="winter"])

shapiro.test(SulProp[Sulseason=="summer"])

shapiro.test(SulProp)

plotNormalHistogram(SulProp[Sulseason=="winter"])

plotNormalHistogram(SulProp[Sulseason=="summer"])

plotNormalHistogram(SulProp)

kruskal.test(SulProp ~ Sulseason, data = Suldata)

boxplot(SulProp~Sulseason)

p<-ggplot(Suldata, aes(x=Sulseason, y=SulProp))+

stat_boxplot(geom='errorbar', linetype=1, width=0.5)+

geom_boxplot()+

# geom_text(x=1, y=155, label="a", size=20)+

#geom_text(x=2, y=155, label="b", size=20)+

xlab("")+

ylab("Gene Abundance")+

#ylim(80,160)+

theme_classic()+

theme(axis.text=element_text(size=30))+

theme(text=element_text(size=40))

p

ggsave("WriteUp/Figs/240828_update/SulfurPhos.svg", width=15, height=10)

rm(betadisper_SIM_funSul,betadisper_SNE_funSul,betadisper_SOR_funSul,

betapart.core_Sulfuns,m6,op,pair_funSulfuns,permutest.betadisper,SIM_funSul,

SNE_funSul,SOR_funSul,SulAbund,Sulfuns,Sulfur,SulProp,Sulseason )

######

#Sulfur Winters

#####

Sulwinter<-subset(Suldata, season=="winter")

Sulwinterfuns<-Sulwinter[15:ncol(Sulwinter)]

Sulyear<-as.factor(Sulwinter$yearseason)

Sulwinterfuns[Sulwinterfuns > 0]<-1

betapart.core_Sulwinter<-betapart.core(Sulwinterfuns)

pair_fun_Sulwinter<-beta.pair(betapart.core_Sulwinter, index.family="sorensen")

SOR_fun_Sulwinter<-(pair_fun_Sulwinter$beta.sor)

SIM_fun_Sulwinter<-(pair_fun_Sulwinter$beta.sim)

SNE_fun_Sulwinter<-(pair_fun_Sulwinter$beta.sne)

betadisper_SOR__Sulwinter <-betadisper(SOR_fun_Sulwinter, Sulyear, type= c("centroid"), bias.adjust=FALSE, add=FALSE)

plot(betadisper_SOR__Sulwinter, axes=c(1,2), cex= 0.7, col = NULL, hull = TRUE, ellipse = FALSE, segments = TRUE, seg.col = "grey", label = FALSE, label.cex = 1, main= "")

(permutest.betadisper<-permutest(betadisper_SOR__Sulwinter, pairwise=FALSE, permutations=999, parallel=getOption("mc.cores"),))

boxplot(betadisper_SOR__Sulwinter, main="")

#non Sig

betadisper_SIM__Sulwinter <-betadisper(SIM_fun_Sulwinter, Sulyear, type= c("centroid"), bias.adjust=FALSE, add=FALSE)

plot(betadisper_SIM__Sulwinter, axes=c(1,2), cex= 0.7, col = NULL, hull = TRUE, ellipse = FALSE, segments = TRUE, seg.col = "grey", label = FALSE, label.cex = 1, main= "")

(permutest.betadisper<-permutest(betadisper_SIM__Sulwinter, pairwise=FALSE, permutations=999, parallel=getOption("mc.cores"),))

boxplot(betadisper_SIM__Sulwinter, main="")

#non Sig

betadisper_SNE__Sulwinter <-betadisper(SNE_fun_Sulwinter, Sulyear, type= c("centroid"), bias.adjust=FALSE, add=FALSE)

plot(betadisper_SNE__Sulwinter, axes=c(1,2), cex= 0.7, col = NULL, hull = TRUE, ellipse = FALSE, segments = TRUE, seg.col = "grey", label = FALSE, label.cex = 1, main= "")

(permutest.betadisper<-permutest(betadisper_SNE__Sulwinter, pairwise=FALSE, permutations=999, parallel=getOption("mc.cores"),))

boxplot(betadisper_SNE__Sulwinter, main="")

#non Sig

adonis2(SOR_fun_Sulwinter~Sulyear, data=Sulwinter, permutations=999, Sulhod='bray')

#non

adonis2(SIM_fun_Sulwinter~Sulyear, data=Sulwinter, permutations=999, Sulhod='bray')

#non

adonis2(SNE_fun_Sulwinter~Sulyear, data=Sulwinter, permutations=999, Sulhod='bray')

#non

SulWinterAbund<-rowSums(Sulwinterfuns)

SulwinterProp<-(SulWinterAbund/(ncol(EPFUfunfunctionRemove)))

shapiro.test(SulwinterProp[Sulyear=="winter1"])

shapiro.test(SulwinterProp[Sulyear=="winter2"])

plotNormalHistogram(SulwinterProp[Sulyear=="winter1"])

plotNormalHistogram(SulwinterProp[Sulyear=="winter2"])

kruskal.test(SulwinterProp ~ Sulyear, data = Sulwinter)

boxplot(SulwinterProp~Sulyear)

p<-ggplot(Sulwinter, aes(x=Sulyear, y=SulWinterAbund))+

stat_boxplot(geom='errorbar', linetype=1, width=0.5)+

geom_boxplot()+

geom_text(x=1, y=155, label="a", size=20)+

geom_text(x=2, y=155, label="b", size=20)+

xlab("")+

ylab("Gene Abundance")+

#ylim(80,160)+

theme_classic()+

theme(axis.text=element_text(size=30))+

theme(text=element_text(size=40))

p

ggsave(".svg", width=15, height=10)

rm(betadisper_SIM__Sulwinter,betadisper_SNE__Sulwinter,betadisper_SOR__Sulwinter,

betapart.core_Sulwinter,m6,Suldata,Sulwinter,SulWinterAbund,Sulwinterfuns,

SulwinterProp,Sulyear, op, p, pair_fun_Sulwinter, permutest.betadisper, SIM_fun_Sulwinter, SNE_fun_Sulwinter,

SOR_fun_Sulwinter)

Supplemental File C Tables and Figures of Results


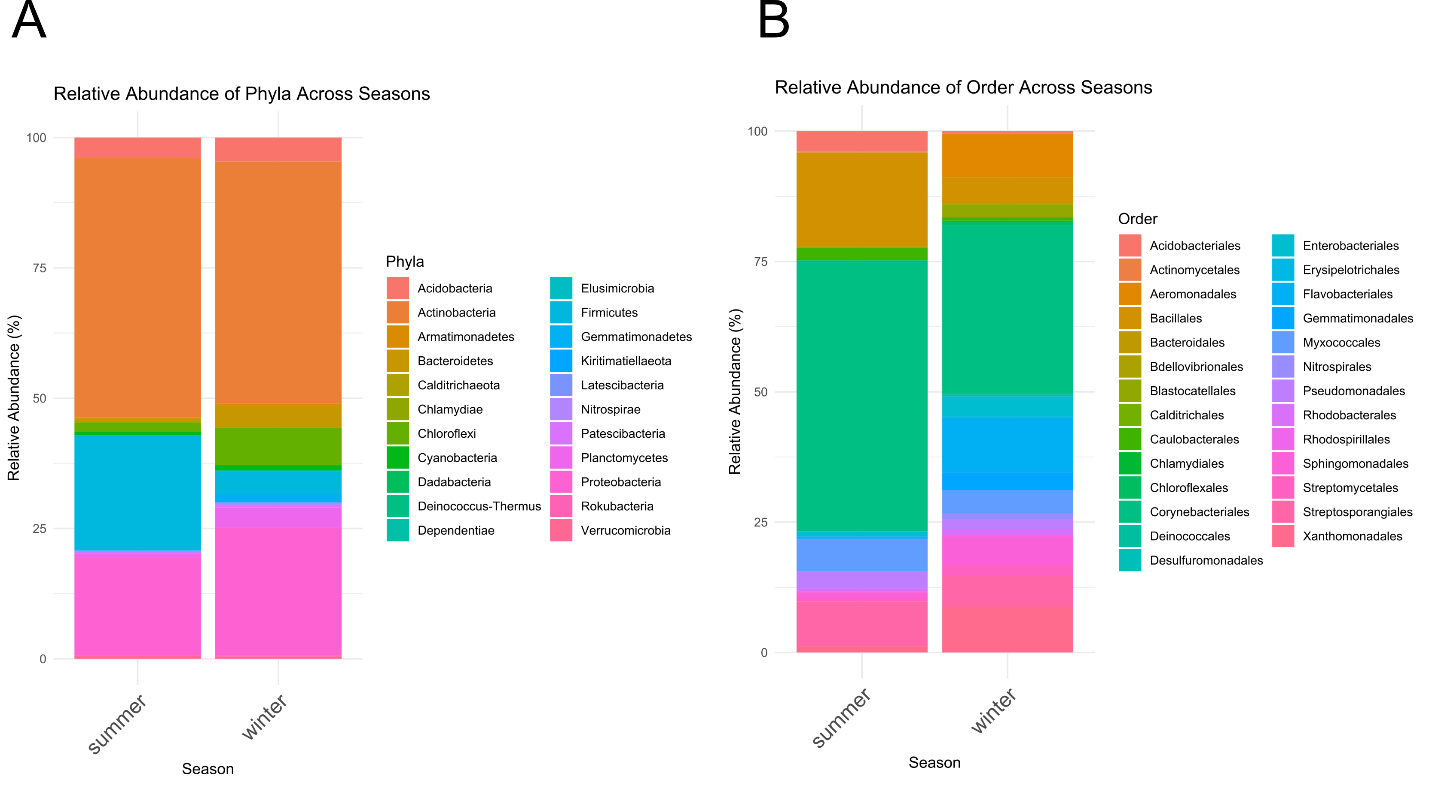


Figure C1. Average relative abundance of A) bacterial Phyla, and B) bacterial order across seasons.

Table C1. Sample metadata. Exact site locations are withheld to protect sensitive locations and species.

| **Sample Code** | **Date** | **Season** | **Site** | **County** | **Level 3 Ecoregion** |
| --- | --- | --- | --- | --- | --- |
| CCB055 | 1/3/2017 | winter | 1 | Cannon | Interior |
| CCB037 | 1/4/2017 | winter | 2 | Bedford | Interior |
| CCB042 | 1/5/2017 | winter | 3 | Sumner | Interior |
| CCB211 | 1/11/2017 | winter | 4 | Campbell | Central Appalachians |
| CCB251 | 2/1/2017 | winter | 5 | Dekalb | Interior |
| CCB063 | 2/2/2017 | winter | 6 | Montgomery | Interior |
| CCB102 | 2/28/2017 | winter | 7 | Dekalb | Interior |
| CCB391 | 7/11/2017 | summer | 8 | Fentress | South West Appalachians |
| CCB396 | 7/25/2017 | summer | 9 | Fentress | South West Appalachians |
| CCB107 | 1/4/2018 | winter | 10 | Van Buren | South West Appalachians |
| CCB583 | 1/5/2018 | winter | 11 | Union | Ridge and Valley |
| CCB571 | 1/5/2018 | winter | 12 | Union | Ridge and Valley |
| CCB725 | 1/12/2018 | winter | 13 | Carter | BlueRidge |
| CCB721 | 1/12/2018 | winter | 14 | Carter | BlueRidge |
| CCB587 | 1/12/2018 | winter | 15 | Sullivan | Ridge and Valley |
| CCB756 | 1/18/2018 | winter | 16 | Jackson | Interior |
| CCB625 | 1/19/2018 | winter | 17 | White | South West Appalachians |
| CCB846 | 1/22/2018 | winter | 18 | Bedford | Interior |
| CCB855 | 1/24/2018 | winter | 19 | Lawrence | Interior |
| CCB596 | 1/30/2018 | winter | 20 | Campbell | Ridge and Valley |
| CCB614 | 1/31/2018 | winter | 21 | Meigs | Ridge and Valley |
| CCB733 | 2/9/2018 | winter | 22 | Grainger | Ridge and Valley |
| CCB1372 | 7/26/2018 | summer | 23 | Franklin | South West Appalachians |
| CCB1104 | 7/26/2018 | summer | 24 | Franklin | South West Appalachians |
| CCB1183 | 7/26/2018 | summer | 25 | Grundy | South West Appalachians |
| CCB1480 | 7/26/2018 | summer | 26 | Franklin | South West Appalachians |

Table C2. Betadisper analysis comparing beta diversity of functions and OTUs measured as multivariate dispersion between summer and winter bats.

| **OTUs** | **p-value** |
| --- | --- |
| Total Beta Diversity | 0.582 |
| Turnover | 0.804 |
| Nestedness | 0.151 |
| **Function** | **p-value** |
| Total Beta Diversity | 0.839 |
| Turnover | 0.912 |
| Nestedness | 0.498 |

Table C3. PERMANOVA analysis comparing average taxonomic (OTU) assemblage structure across season.

|  | **Df** | **Sum of Squares** | **F test** | **R^2^** | ***P* value** |
| --- | --- | --- | --- | --- | --- |
| **Total Beta Diversity** | | | | | |
| Season | 1 | 0.7295 | 1.8388 | 0.07116 | 0.001* |
| Residual | 24 | 9.5212 |  | 0.92884 |  |
| Total | 25 | 10.2507 |  | 1 |  |
| **Turnover** | | | | | |
| Season | 1 | 0.6731 | 1.823 | 0.07059 | 0.001* |
| Residual | 24 | 8.8613 |  | 0.92941 |  |
| Total | 25 | 9.5344 |  | 1 |  |
| **Nestedness** | | | | | |
| Season | 1 | 0.0014048 | 1.3547 | 0.05343 | 0.377 |
| Residual | 24 | 0.0248875 |  | 0.94657 |  |
| Total | 25 | 0.0262923 |  | 1 |  |

Table C4. Betadisper analysis comparing beta diversity of functions and OTUs measured as multivariate dispersion across winters.

| **OTUs** | **p-value** |
| --- | --- |
| Total Beta Diversity | 0.153 |
| Turnover | 0.083 |
| Nestedness | 0.378 |
| **Function** | **p-value** |
| Total Beta Diversity | 0.435 |
| Turnover | 0.732 |
| Nestedness | 0.564 |

Table C5. PERMANOVA analysis comparing average winter taxonomic (OTU) assemblage structure across year.

|  | **Df** | **Sum of Squares** | **F test** | **R^2^** | ***P* value** |
| --- | --- | --- | --- | --- | --- |
| **Total Beta Diversity** | | | | | |
| Year | 1 | 0.4035 | 1.0453 | 0.05489 | 0.26 |
| Residual | 18 | 6.9488 |  | 0.94511 |  |
| Total | 19 | 7.3524 |  | 1 |  |
| **Turnover** | | | | | |
| Year | 1 | 0.3969 | 1.1225 | 0.0587 | 0.187 |
| Residual | 18 | 6.3641 |  | 0.9413 |  |
| Total | 19 | 6.7610 |  | 1 |  |
| **Nestedness** | | | | | |
| Year | 1 | 0.0008083 | 0.6189 | 0.03324 | 0.529 |
| Residual | 18 | 0.0235071 |  | 0.96676 |  |
| Total | 19 | 0.0243155 |  | 1 |  |

Table C6. GLM comparing the fraction of used OTUs in the prediction of assemblage function between summer and winter bats

| **FTU between season** |
| --- |
| Formula: FTU ~ seasonTax, family = binomial, data = EPFU |

| Deviance Residuals: |  |  |  |  |
| --- | --- | --- | --- | --- |
| **Min** | **1Q** | **Median** | **3Q** | **Max** |
| -0.35527 | -0.12053 | 0.00501 | 0.13046 | 0.28340 |
|  |  |  |  |  |
| **Coefficients:** | **Estimate** | **Std Error** | **z value** | **Pr(>\|z\|)** |
| (Intercept) | 0.8343 | 0.8886 | 0.939 | 0.348 |
| Season | 0.4682 | 1.0426 | 0.449 | 0.653 |
| Null deviance: 0.87137 on 25 degrees of freedom | | | | |
| Residual deviance: 0.67571 on 24 degrees of freedom | | | | |
| AIC: 17.946 |  |  |  |  |

Table C7. PERMANOVA analysis comparing average functional assemblage structure across season.

|  | **Df** | **Sum of Squares** | **F test** | **R^2^** | ***P* value** |
| --- | --- | --- | --- | --- | --- |
| **Total Beta Diversity** | | | | | |
| Season | 1 | 0.002595 | 1.0115 | 0.04044 | 0.359 |
| Residual | 24 | 0.061570 |  | 0.95956 |  |
| Total | 25 | 0.064165 |  | 1 |  |
| **Turnover** | | | | | |
| Season | 1 | 0.0019895 | 2.1803 | 0.08328 | 0.166 |
| Residual | 24 | 0.0218992 |  | 0.91672 |  |
| Total | 25 | 0.0238887 |  | 1 |  |
| **Nestedness** | | | | | |
| Season | 1 | -0.0000077 | -0.0116 | -0.00048 | 0.926 |
| Residual | 24 | 0.0160162 |  | 1.00048 |  |
| Total | 25 | 0.0160085 |  | 1 |  |

Table C8. PERMANOVA analysis comparing average functional assemblage structure across years

|  | **Df** | **Sum of Squares** | **F test** | **R^2^** | ***P* value** |
| --- | --- | --- | --- | --- | --- |
| **Total Beta Diversity** | | | | | |
| Year | 1 | 0.002925 | 1.269 | 0.06586 | 0.21 |
| Residual | 18 | 0.041490 |  | 0.93414 |  |
| Total | 19 | 0.044415 |  | 1 |  |
| **Turnover** | | | | | |
| Year | 1 | 0.0021684 | 2.74607 | 0.13298 | 0.087 |
| Residual | 18 | 0.0141377 |  | 0.86702 |  |
| Total | 19 | 0.0163061 |  | 1 |  |
| **Nestedness** | | | | | |
| Year | 1 | -0.0000168 | -0.0264 | -0.00149 | 0.933 |
| Residual | 18 | 0.0114169 |  | 1.00147 |  |
| Total | 19 | 0.0114002 |  | 1 |  |

Table C9. Betadisper analysis comparing beta diversity of select functional pathways measured as multivariate dispersion across seasons. Pathways include Membrane Transport, Metabolism of Terpenoids and Polyketides (MTP), Biosynthesis of Secondary Metabolites (BSM), Glycolysis, Methan metabolism, Sulfur metabolism, and Oxidative Phosphorylation.

| **Membrane Transport** | **Season p-value** | **Year p-value** |
| --- | --- | --- |
| Total Beta Diversity | 0.688 | 0.28 |
| Turnover | 0.574 | 0.484 |
| Nestedness | 0.273 | 0.355 |
| **MTP** | **p-value** | **p-value** |
| Total Beta Diversity | 0.173 | 0.111 |
| Turnover | 0.13 | 0.876 |
| Nestedness | 0.733 | 0.354 |
| **BSM** | **p-value** | **p-value** |
| Total Beta Diversity | 0.329 | 0.396 |
| Turnover | 0.455 | 0.278 |
| Nestedness | 0.778 | 0.32 |
| **Glycolysis** | **p-value** | **p-value** |
| Total Beta Diversity | 0.55 | 0.535 |
| Turnover | 0.401 | 0.718 |
| Nestedness | 0.717 | 0.738 |
| **Methane** | **p-value** | **p-value** |
| Total Beta Diversity | 0.944 | 0.886 |
| Turnover | 0.846 | 0.273 |
| Nestedness | 0.928 | 0.593 |
| **Sulfur** | **p-value** | **p-value** |
| Total Beta Diversity | 0.686 | 0.167 |
| Turnover | 0.519 | 0.357 |
| Nestedness | 0.392 | 0.514 |
| **Oxidative Phosphorylation** | **p-value** | **p-value** |
| Total Beta Diversity | 0.109 | 0.804 |
| Turnover | 0.178 | 0.625 |
| Nestedness | 0.302 | 0.408 |

Table C10. PERMANOVA analysis comparing average functional assemblage structure for Membrane Transport genes across seasons

|  | **Df** | **Sum of Squares** | **F test** | **R^2^** | ***P* value** |
| --- | --- | --- | --- | --- | --- |
| **Total Beta Diversity** | | | | | |
| Season | 1 | 0.0006487 | 0.8389 | 0.03377 | 0.517 |
| Residual | 24 | 0.0185591 |  | 0.96623 |  |
| Total | 25 | 0.0192078 |  | 1 |  |
| **Turnover** | | | | | |
| Season | 1 | 0.0004754 | 2.2595 | 0.8605 | 0.163 |
| Residual | 24 | 0.0050500 |  | 0.91395 |  |
| Total | 25 | 0.0055254 |  | 1 |  |
| **Nestedness** | | | | | |
| Season | 1 | 0.0000338 | 0.1165 | 0.00483 | 0.731 |
| Residual | 24 | 0.0069616 |  | 0.99517 |  |
| Total | 25 | 0.0069954 |  | 1 |  |

Table C11. PERMANOVA analysis comparing average functional assemblage structure for Membrane Transport genes across years

|  | **Df** | **Sum of Squares** | **F test** | **R^2^** | ***P* value** |
| --- | --- | --- | --- | --- | --- |
| **Total Beta Diversity** | | | | | |
| Year | 1 | 0.0006996 | 0.8477 | 0.04497 | 0.535 |
| Residual | 18 | 0.0148566 |  | 0.95503 |  |
| Total | 19 | 0.0155562 |  | 1 |  |
| **Turnover** | | | | | |
| Year | 1 | 0.0003198 | 1.7221 | 0.08732 | 0.346 |
| Residual | 18 | 0.0033428 |  | 0.91268 |  |
| Total | 19 | 0.0036626 |  | 1 |  |
| **Nestedness** | | | | | |
| Year | 1 | 0.0000338 | 0.2345 | 0.01286 | 0.658 |
| Residual | 18 | 0.0069616 |  | 0.98714 |  |
| Total | 19 | 0.0069954 |  | 1 |  |

Table C12. PERMANOVA analysis comparing average functional assemblage structure for Metabolism of terpenoids and polyketides genes across seasons

|  | **Df** | **Sum of Squares** | **F test** | **R^2^** | ***P* value** |
| --- | --- | --- | --- | --- | --- |
| **Total Beta Diversity** | | | | | |
| Season | 1 | 0.006698 | 1.4349 | 0.05641 | 0.214 |
| Residual | 24 | 0.112028 |  | 0.94359 |  |
| Total | 25 | 0.118725 |  | 1 |  |
| **Turnover** | | | | | |
| Season | 1 | 0.0004754 | 2.2595 | 0.8605 | 0.163 |
| Residual | 24 | 0.0050500 |  | 0.91395 |  |
| Total | 25 | 0.0055254 |  | 1 |  |
| **Nestedness** | | | | | |
| Season | 1 | 0.0000338 | 0.1165 | 0.00483 | 0.731 |
| Residual | 24 | 0.0069616 |  | 0.99517 |  |
| Total | 25 | 0.0069954 |  | 1 |  |

Table C13. PERMANOVA analysis comparing average functional assemblage structure for Metabolism of terpenoids and polyketides genes across years

|  | **Df** | **Sum of Squares** | **F test** | **R^2^** | ***P* value** |
| --- | --- | --- | --- | --- | --- |
| **Total Beta Diversity** | | | | | |
| Year | 1 | 0.007041 | 1.5962 | 0.08145 | 0.149 |
| Residual | 18 | 0.079404 |  | 0.91855 |  |
| Total | 19 | 0.086445 |  | 1 |  |
| **Turnover** | | | | | |
| Year | 1 | 0.0010015 | 1.1076 | 0.05797 | 0.507 |
| Residual | 18 | 0.0162762 |  | 0.94203 |  |
| Total | 19 | 0.0172777 |  | 1 |  |
| **Nestedness** | | | | | |
| Year | 1 | 0.004327 | 2.2222 | 0.10989 | 0.157 |
| Residual | 18 | 0.035047 |  | 0.89011 |  |
| Total | 19 | 0.039373 |  | 1 |  |

Table C14. PERMANOVA analysis comparing average functional assemblage structure for Biosynthesis of secondary metabolism genes across seasons

|  | **Df** | **Sum of Squares** | **F test** | **R^2^** | ***P* value** |
| --- | --- | --- | --- | --- | --- |
| **Total Beta Diversity** | | | | | |
| Season | 1 | 0.002907 | 1.0909 | 0.04348 | 0.331 |
| Residual | 24 | 0.063964 |  | 0.95652 |  |
| Total | 25 | 0.066871 |  | 1 |  |
| **Turnover** | | | | | |
| Season | 1 | 0.0000673 | 0.0661 | 0.00275 | 0.858 |
| Residual | 24 | 0.0244311 |  | 0.99725 |  |
| Total | 25 | 0.0244983 |  | 1 |  |
| **Nestedness** | | | | | |
| Season | 1 | 0.0011437 | 1.6237 | 0.06337 | 0.197 |
| Residual | 24 | 0.0169046 |  | 0.93663 |  |
| Total | 25 | 0.0180483 |  | 1 |  |

Table C15. PERMANOVA analysis comparing average functional assemblage structure for Biosynthesis of secondary metabolism genes across years

|  | **Df** | **Sum of Squares** | **F test** | **R^2^** | ***P* value** |
| --- | --- | --- | --- | --- | --- |
| **Total Beta Diversity** | | | | | |
| Year | 1 | 0.003167 | 1.3036 | 0.06753 | 0.204 |
| Residual | 18 | 0.043725 |  | 0.93247 |  |
| Total | 19 | 0.046892 |  | 1 |  |
| **Turnover** | | | | | |
| Year | 1 | 0.0009746 | 1.1473 | 0.05992 | 0.443 |
| Residual | 18 | 0.0152899 |  | 0.94008 |  |
| Total | 19 | 0.0162645 |  | 1 |  |
| **Nestedness** | | | | | |
| Year | 1 | 0.0016405 | 2.4305 | 0.11896 | 0.142 |
| Residual | 18 | 0.0121496 |  | 0.88104 |  |
| Total | 19 | 0.0137901 |  | 1 |  |

Table C16. PERMANOVA analysis comparing average functional assemblage structure for Glycolysis genes across seasons.

|  | **Df** | **Sum of Squares** | **F test** | **R^2^** | ***P* value** |
| --- | --- | --- | --- | --- | --- |
| **Total Beta Diversity** | | | | | |
| Season | 1 | 0.0002733 | 0.3452 | 0.01418 | 0.789 |
| Residual | 24 | 0.0190001 |  | 0.98582 |  |
| Total | 25 | 0.0192734 |  | 1 |  |
| **Turnover** | | | | | |
| Season | 1 | 0.0002198 | 1.0792 | 0.04303 | 0.472 |
| Residual | 24 | 0.0048887 |  | 0.95697 |  |
| Total | 25 | 0.0051085 |  | 1 |  |
| **Nestedness** | | | | | |
| Season | 1 | 0.0000259 | 0.0715 | 0.00297 | 0.777 |
| Residual | 24 | 0.0086957 |  | 0.99703 |  |
| Total | 25 | 0.0087216 |  | 1 |  |

Table C17. PERMANOVA analysis comparing average functional assemblage structure for Glycolysis genes across years

|  | **Df** | **Sum of Squares** | **F test** | **R^2^** | ***P* value** |
| --- | --- | --- | --- | --- | --- |
| **Total Beta Diversity** | | | | | |
| Year | 1 | 0.0000418 | 0.0478 | 0.00265 | 0.937 |
| Residual | 18 | 0.0157284 |  | 0.99735 |  |
| Total | 19 | 0.0157702 |  | 1 |  |
| **Turnover** | | | | | |
| Year | 1 | 0.0004072 | 1.8482 | 0.09311 | 0.357 |
| Residual | 18 | 0.0039661 |  | 0.90689 |  |
| Total | 19 | 0.0043733 |  | 1 |  |
| **Nestedness** | | | | | |
| Year | 1 | 0.0000343 | 0.0866 | 0.00479 | 0.749 |
| Residual | 18 | 0.0071313 |  | 0.99521 |  |
| Total | 19 | 0.0071656 |  | 1 |  |

Table C18. PERMANOVA analysis comparing average functional assemblage structure for Oxidative Phosphorylation genes across seasons.

|  | **Df** | **Sum of Squares** | **F test** | **R^2^** | ***P* value** |
| --- | --- | --- | --- | --- | --- |
| **Total Beta Diversity** | | | | | |
| Season | 1 | 0.001689 | 0.9285 | 0.03725 | 0.491 |
| Residual | 24 | 0.043657 |  | 0.96275 |  |
| Total | 25 | 0.045346 |  | 1 |  |
| **Turnover** | | | | | |
| Season | 1 | 0.000905 | 1.8763 | 0.07251 | 0.31 |
| Residual | 24 | 0.011571 |  | 0.92749 |  |
| Total | 25 | 0.012475 |  | 1 |  |
| **Nestedness** | | | | | |
| Season | 1 | 4.81E-05 | 0.0603 | 0.00251 | 0.772 |
| Residual | 24 | 0.019131 |  | 0.99749 |  |
| Total | 25 | 0.019179 |  | 1 |  |

Table C19. PERMANOVA analysis comparing average functional assemblage structure for Oxidative Phosphorylation genes across years.

|  | **Df** | **Sum of Squares** | **F test** | **R^2^** | ***P* value** |
| --- | --- | --- | --- | --- | --- |
| **Total Beta Diversity** | | | | | |
| Year | 1 | 0.000861 | 0.03171 | 0.5894 | 0.664 |
| Residual | 18 | 0.026299 | 0.96829 |  |  |
| Total | 19 | 0.02716 | 1 |  |  |
| **Turnover** | | | | | |
| Year | 1 | 0.000135 | 0.02264 | 0.4169 | 0.584 |
| Residual | 18 | 0.005841 | 0.97736 |  |  |
| Total | 19 | 0.005977 | 1 |  |  |
| **Nestedness** | | | | | |
| Year | 1 | 0.000214 | 0.01713 | 0.3138 | 0.593 |
| Residual | 18 | 0.012274 | 0.98287 |  |  |
| Total | 19 | 0.012488 | 1 |  |  |

Table C20. PERMANOVA analysis comparing average functional assemblage structure for Methane metabolism genes across seasons.

|  | **Df** | **Sum of Squares** | **F test** | **R^2^** | ***P* value** |
| --- | --- | --- | --- | --- | --- |
| **Total Beta Diversity** | | | | | |
| Season | 1 | 0.003022 | 0.02171 | 0.5327 | 0.771 |
| Residual | 24 | 0.136149 | 0.97829 |  |  |
| Total | 25 | 0.139171 | 1 |  |  |
| **Turnover** | | | | | |
| Season | 1 | 0.002227 | 0.06561 | 1.6853 | 0.309 |
| Residual | 24 | 0.031718 | 0.93439 |  |  |
| Total | 25 | 0.033946 | 1 |  |  |
| **Nestedness** | | | | | |
| Season | 1 | 0.000073 | 0.00136 | 0.0328 | 0.841 |
| Residual | 24 | 0.053439 | 0.99864 |  |  |
| Total | 25 | 0.053512 | 1 |  |  |

Table C21. PERMANOVA analysis comparing average functional assemblage structure for Methane metabolism genes across years.

|  | **Df** | **Sum of Squares** | **F test** | **R^2^** | ***P* value** |
| --- | --- | --- | --- | --- | --- |
| **Total Beta Diversity** | | | | | |
| Year | 1 | 0.004648 | 0.04385 | 0.8255 | 0.556 |
| Residual | 18 | 0.101342 | 0.95615 |  |  |
| Total | 19 | 0.10599 | 1 |  |  |
| **Turnover** | | | | | |
| Year | 1 | -0.00018 | -0.0068 | -0.1216 | 0.789 |
| Residual | 18 | 0.027053 | 1.0068 |  |  |
| Total | 19 | 0.02687 | 1 |  |  |
| **Nestedness** | | | | | |
| Year | 1 | 0.000852 | 0.02077 | 0.3817 | 0.527 |
| Residual | 18 | 0.040176 | 0.97923 |  |  |
| Total | 19 | 0.041029 | 1 |  |  |

Table C22. PERMANOVA analysis comparing average functional assemblage structure for Sulfur metabolism genes across seasons.

|  | **Df** | **Sum of Squares** | **F test** | **R^2^** | ***P* value** |
| --- | --- | --- | --- | --- | --- |
| **Total Beta Diversity** | | | | | |
| Season | 1 | 0.001348 | 0.02266 | 0.5565 | 0.721 |
| Residual | 24 | 0.058123 | 0.97734 |  |  |
| Total | 25 | 0.059471 | 1 |  |  |
| **Turnover** | | | | | |
| Season | 1 | 9.05E-05 | 0.00606 | 0.1462 | 0.695 |
| Residual | 24 | 0.014856 | 0.99394 |  |  |
| Total | 25 | 0.014947 | 1 |  |  |
| **Nestedness** | | | | | |
| Season | 1 | 7.87E-05 | 0.00289 | 0.0696 | 0.795 |
| Residual | 24 | 0.027126 | 0.99711 |  |  |
| Total | 25 | 0.027204 | 1 |  |  |

Table C23. PERMANOVA analysis comparing average functional assemblage structure for Sulfur metabolism genes across years.

|  | **Df** | **Sum of Squares** | **F test** | **R^2^** | ***P* value** |
| --- | --- | --- | --- | --- | --- |
| **Total Beta Diversity** | | | | | |
| Year | 1 | 0.001843 | 0.04241 | 0.7972 | 0.556 |
| Residual | 18 | 0.041616 | 0.95759 |  |  |
| Total | 19 | 0.043459 | 1 |  |  |
| **Turnover** | | | | | |
| Year | 1 | 0.000944 | 0.08119 | 1.5905 | 0.302 |
| Residual | 18 | 0.010682 | 0.91881 |  |  |
| Total | 19 | 0.011625 | 1 |  |  |
| **Nestedness** | | | | | |
| Year | 1 | 0.000136 | 0.00683 | 0.1238 | 0.761 |
| Residual | 18 | 0.019713 | 0.99317 |  |  |
| Total | 19 | 0.019849 | 1 |  |  |

Table C24. Comparison of relative abundance of genes within pathways hypothesized to vary across seasons and years.

| **Pathway** | **Season p-value** | **Year p-value** |
| --- | --- | --- |
| Membrane Transport | 0.903 | 0.8365 |
| MTP | 0.113 | 0.164 |
| BSM | 0.204 | 0.149 |
| Glycolysis | 0.7822 | 0.856 |
| Methane | 0.7598 | 0.171 |
| Sulfur | 0.8062 | 0.9336 |
| Oxidative Phosphorylation | 0.4632 | 0.577 |
